# MicroRNA-125b upregulation confers aromatase inhibitor resistance and is a novel marker of poor prognosis in breast cancer

**DOI:** 10.1186/s13058-015-0515-1

**Published:** 2015-01-30

**Authors:** Paul Vilquin, Caterina F Donini, Marie Villedieu, Evelyne Grisard, Laura Corbo, Thomas Bachelot, Julie A Vendrell, Pascale A Cohen

**Affiliations:** ISPB, Faculté de Pharmacie, 8 Avenue Rockefeller, 69008 Lyon, France; Université Lyon 1, 8 Avenue Rockefeller, 69008 Lyon, France; INSERM U1052, CNRS UMR5286, Centre de Recherche en Cancérologie de Lyon, 28 Rue Laennec, 69008 Lyon, France; Unité Cancer et Environnement, Centre Léon Bérard–Université Lyon 1, 28 Rue Laennec, 69008 Lyon, France; Centre Léon Bérard, 28 Rue Laennec, 69008 Lyon, France; ProfileXpert, SFR Lyon-Est, 69008 Lyon, France; ISPBL–Faculté de Pharmacie de Lyon, 8 Avenue Rockefeller, 69373 Lyon, Cedex 08 France

## Abstract

**Introduction:**

Increasing evidence indicates that microRNAs (miRNAs) are important players in oncogenesis. Considering the widespread use of aromatase inhibitors (AIs) in endocrine therapy as a first-line treatment for postmenopausal estrogen receptor α–positive breast cancer patients, identifying deregulated expression levels of miRNAs in association with AI resistance is of utmost importance.

**Methods:**

To gain further insight into the molecular mechanisms underlying the AI resistance, we performed miRNA microarray experiments using a new model of acquired resistance to letrozole (Res-Let cells), obtained by long-term exposure of aromatase-overexpressing MCF-7 cells (MCF-7aro cells) to letrozole, and a model of acquired anastrozole resistance (Res-Ana cells). Three miRNAs (miR-125b, miR-205 and miR-424) similarly deregulated in both AI-resistant cell lines were then investigated in terms of their functional role in AI resistance development and breast cancer cell aggressiveness and their clinical relevance using a cohort of 65 primary breast tumor samples.

**Results:**

We identified the deregulated expression of 33 miRNAs in Res-Let cells and of 18 miRNAs in Res-Ana cells compared with the sensitive MCF-7aro cell line. The top-ranked Kyoto Encyclopedia of Genes and Genomes pathways delineated by both miRNA signatures converged on the AKT/mTOR pathway, which was found to be constitutively activated in both AI-resistant cell lines. We report for the first time, to our knowledge, that ectopic overexpression of either miR-125b or miR-205, or the silencing of miR-424 expression, in the sensitive MCF-7aro cell line was sufficient to confer resistance to letrozole and anastrozole, to target and activate the AKT/mTOR pathway and to increase the formation capacity of stem-like and tumor-initiating cells possessing self-renewing properties. Increasing miR-125b expression levels was also sufficient to confer estrogen-independent growth properties to the sensitive MCF-7aro cell line. We also found that elevated miR-125b expression levels were a novel marker for poor prognosis in breast cancer and that targeting miR-125b in Res-Let cells overcame letrozole resistance.

**Conclusion:**

This study highlights that acquisition of specific deregulated miRNAs is a newly discovered alternative mechanism developed by AI-resistant breast cancer cells to achieve constitutive activation of the AKT/mTOR pathway and to develop AI resistance. It also highlights that miR-125b is a new biomarker of poor prognosis and a candidate therapeutic target in AI-resistant breast cancers.

**Electronic supplementary material:**

The online version of this article (doi:10.1186/s13058-015-0515-1) contains supplementary material, which is available to authorized users.

## Introduction

In approximately 75% of postmenopausal patients, breast cancer is a hormone-dependent disease that relies on the mitogenic effects of estrogen to drive carcinogenesis. Endocrine therapies, including estrogen receptor α (ERα) modulators and aromatase inhibitors (AIs), are the most suitable treatment for ERα-positive (ER+) breast cancer patients. Recently, nonsteroidal AIs (for example, letrozole, anastrozole) that block the biosynthesis of estrogens have proven more effective than the selective estrogen receptor modulator tamoxifen (Tam) in the treatment of postmenopausal patients with ER+ breast cancer [1]. Despite the demonstrated clinical efficacy of AIs, however, *de novo* and acquired resistance still occurs and constitutes a major impediment to successful therapy.

At present, acquired resistance to endocrine therapy is considered to be a progressive, stepwise phenomenon whereby breast cancer cells are converted from an estrogen-dependent phenotype, which is responsive to endocrine therapy, to a nonresponsive phenotype and eventually to an estrogen-independent phenotype. Among the molecular mechanisms involved in the acquisition of endocrine resistance, a switch from steroid signaling to growth factor signaling pathways has been the focus of recent studies, which have demonstrated the activation of the phosphatidylinositol 3-kinase (PI3K)/AKT/mammalian target of rapamycin (mTOR) and/or mitogen-activated protein kinase (MAPK) pathways, both in breast cancer cell lines and in breast tumors [2-8]. Activation of these survival pathways may contribute to endocrine resistance via the activation of kinases in an ER-dependent [9] as well as ER-independent fashion [2,10].

MicroRNAs (miRNAs) are short, noncoding RNAs that generally base pair within the 3′ untranslated (3′UTR) region of target mRNAs, causing translational inhibition and/or mRNA degradation. A growing body of evidence favors miRNAs’ being important players in oncogenesis [11], with some able to act as oncogenes, others as tumor suppressors and others displaying either oncogenic or tumor-suppressive activities, depending on the tissue and tumor context [11-13]. Widespread deregulated expression of miRNAs would thus be expected to represent another hallmark of cancer, providing not only biomarkers but also novel therapeutic targets. Recent investigations have revealed that miRNAs are involved in the development of drug resistance, but little is known about the miRNA-driven molecular mechanisms governing the drug-resistant signal transduction network [14,15].

Increasing amounts of data support an involvement of miRNAs in estrogen action and/or in endocrine resistance, with most studies dedicated to Tam or fulvestrant resistance. A close cross-talk appears to exist between ERα and specific miRNAs, with several miRNAs found to regulate ERα, which conversely negatively regulates the expression of some miRNAs [16]. Complexity was further revealed by the observation that miRNAs are also able to regulate ERα activity by repressing the expression of ERα transcriptional cofactors [16]. Furthermore, some miRNA signatures have been identified by microarray analysis in Tam- or fulvestrant-resistant breast cancer cell lines [17,18]. Several miRNAs, such as the miR-200 family [19], miR-375 [20], miR-221/222 [21,22], miR-15a/16 [23], miR-101 and miR-519a [24,25], have been shown to regulate molecular targets or functional signaling pathways associated with Tam or fulvestrant resistance. To date, only one study research team has investigated miRNA expression profiles associated with AI resistance [26]. They found that miR-128a, previously identified as being associated with breast cancer aggressiveness [27], was highly expressed in letrozole-resistant breast cancer cells compared with their sensitive counterpart and targeted the transforming growth factor β (TGF-β) signaling pathway.

Bearing in mind that any one given miRNA can have several targets, some belonging to the same functional network or signaling pathway, and that the 3′UTR of a single gene is frequently targeted by several different miRNAs, our primary aim in this study was to capture a global view of the miRNA expression profiles associated with AI resistance in the hope of identifying common miRNA-targeted specific functional networks. Our second aim was to select the most relevant miRNAs that represent candidate biomarkers or putative therapeutic targets of ER+ breast cancers treated by AIs. In this study, we performed a large-scale investigation of miRNA expression profiles associated with letrozole or anastrozole resistance using two *in vitro* models of AI-acquired resistance. We report the acquisition of several deregulated miRNAs as a newly discovered alternative mechanism developed by the AI-resistant breast cancer cells to achieve constitutive activation of the AKT/mTOR pathway and to develop AI resistance. We also demonstrate, for the first time to our knowledge, that elevated miR-125b expression levels constitute a novel marker for poor prognosis in breast cancer and that targeting miR-125b in letrozole-resistant cells overcame letrozole resistance.

## Methods

### Establishment of resistant cell lines and culture conditions

A new letrozole-resistant cell line, denoted Res-Let, was established from the ER+ MCF-7-derived breast cancer cell line stably transfected with the human aromatase gene (MCF-7aro cells, kindly provided by Dr Shiuan Chen) [28]. The MCF-7aro cells were exposed during a 20-week period to increasing concentrations (1, 3 and 5 μM) of letrozole (Novartis, Basel, Switzerland) in Dulbecco’s modified Eagle’s medium without phenol red, supplemented with 3% steroid-depleted, dextran-coated, charcoal-treated fetal calf serum (DCC medium) containing 25 nM of 4-androstenedione (AD) (Sigma-Aldrich, St Louis, MO, USA). The previously described Res-Ana cells [6] were used as a model of acquired resistance to anastrozole. The cells were purged in DCC medium for 4 days prior to each experiment described below, then treated with 25 nM AD combined, or not, with the appropriate treatment. Media and treatments were changed every 2 days.

### Cytotoxicity assay

A total of 10^4^ cells/well were plated in a 96-well plate and treated for 4 days with 25 nM of AD combined with letrozole, anastrozole (AstraZeneca, London, UK) or MK-2206 (Merck Sharp & Dohme, Whitehouse Station, NJ, USA). Cell viability was assessed as previously described [29].

### Total RNA extraction

Total RNA was prepared from cell lines or tumor samples using the miRNeasy Mini Kit (Qiagen, Hilden, Germany). RNA integrity was checked using the BioAnalyzer 2100 (Agilent Technologies, Palo Alto, CA, USA).

### GeneChip miRNA 3.0 Array Affymetrix experiment and analysis

For MCF-7aro, Res-Let or Res-Ana cells, two independent cell culture replicates were used to generate total RNA. Complex probes were produced from total RNA using the FlashTag™ Biotin HSR RNA labeling kit (Affymetrix, Santa Clara, CA, USA) and then hybridized to each GeneChip miRNA 3.0 array according to the manufacturer’s recommendations (Affymetrix). Experiments were performed by the ProfileXpert platform (Lyon, France). Scanned images of microarray chips were analyzed using Expression Console software (Affymetrix) with the default settings. Raw data were processed using different algorithms. In particular, the detection above background algorithm and the robust multiarray average background adjustment algorithm were performed to remove the background value. For each cell culture replicate, expression values were given as fold changes (FCs) corresponding to the ratio of the value for resistant cells to that for MCF-7aro cells. Only probe sets with FC values superior to the selected cutoff (greater than or equal to 1.7 or less than or equal to −1.7) in the two independent cell culture replicates were considered to be differentially expressed. The microarray data have been deposited in the Gene Expression Omnibus (GEO) database [GEO:GSE43766].

### Bioinformatic analysis

Expression profiles of the deregulated miRNAs were visualized using the Cluster and TreeView software programs [30]. The list of deregulated miRNAs was then submitted to the DIANA-mirPath algorithm, using the TargetScan 5 search algorithm, to find specific pathways that may be altered by miRNA modulations [31]. The miRWalk database was used to identify the miRNAs targeting the AKT pathway [32].

### Real-time quantitative PCR

Reverse transcription was carried out using the Universal cDNA Synthesis Kit II (Exiqon, Vedbæk, Denmark). Quantitative real-time PCR (RTQ-PCR) measurements were performed using the ExiLENT SYBR Green Master Mix with commercially available primers for miRNAs (miR-125b-5p, miR-205-5p and miR-424-3p) as recommended by the manufacturer (Exiqon). U6 small nuclear RNA was used as an endogenous control for normalization [33].

### Transfection of miRNA oligonucleotides

MCF-7aro cells were transfected with 5 nM of miR-125b-5p, miR-205-5p or miR-424-3p mirVana miRNA mimics (Life Technologies, Carlsbad, CA, USA) or with 5 nM of miR-424-3p, miR-125b-5p or miR-205-5p inhibitors (Exiqon) or corresponding negative controls using Lipofectamine RNAiMAX transfection reagent (Life Technologies). RNA extraction, RTQ-PCR experiments, Western blot analysis and cytotoxicity assays using cells transfected with miRNA mimics or inhibitors were performed 72 hours posttransfection.

### Mammosphere assay

After transfection, or not, with miRNA oligonucleotides, single-cell suspensions were seeded using nonadherent mammosphere culture conditions [34]. After 7 days, primary mammospheres (PMs) were counted, collected, trypsinized, transfected again with miRNA oligonucleotides and replated for 10 days in nonadherent culture conditions to generate second-generation mammospheres. The culture media were replenished every 2 to 3 days.

### Proliferation analysis in estrogen-free conditions

A total of 3 × 10^4^ cells/well were plated in a 24-well plate and grown for 2, 3.5 or 6 days in steroid-free medium before counting.

### Western blot analysis

Western blot experiments were performed as previously described [35]. The antibodies used were anti-phospho-Ser473-AKT (clone 587 F11, 1:1,000 dilution), anti-AKT (clone C67E7, 1:1,000 dilution), anti-phospho-Ser9-GSK-3β (catalog number 9331, 1:1,000 dilution), anti-GSK3β (clone 27C10, 1:1,000 dilution), anti-phospho-thr389-p70S6K (clone E10, 1:1,000 dilution), anti-p70S6K (clone 49D7, 1:1,000 dilution) (all from Cell Signaling Technology, Beverly, MA, USA) and anti-α-tubulin (clone DM1A, 1:5,000; Sigma-Aldrich).

### Breast tumor cohorts

Women with primary breast tumors and known clinical follow-up who had not received any therapy before surgery and who relapsed, or not, while receiving endocrine therapy and/or chemotherapy were recruited from the BB-0033-00050 Centre de Ressources Biologiques of the Centre Léon Bérard (Lyon, France) (Additional file 1: Table S1). Informed consent was obtained from all patients, and the study was approved by the center’s ethics committee. It was checked that the average relapse-free survival (RFS) and overall survival of the cohort used in this study were similar to those of a larger breast cancer cohort (*N* = 2,978) [36,37]. Our cohort was subdivided into breast cancer subtypes according to the St Gallen recommendation [38] using the immunohistological markers ER, progesterone receptor (PR), human epidermal growth factor receptor 2 (HER2)/neu/ERBB2 (receptor tyrosine kinase) and the Scarff-Bloom-Richardson (SBR) grade (indicative of proliferation) as follows: HR+ (ER+ and/or PR+), luminal A (ER+ and/or PR+, HER2−, low proliferation (SBR1 or SBR2)) or luminal B (ER+ and/or PR+, HER2+ and/or SBR3 (high proliferation)). IBM SPSS software (IBM, Armonk, NY, USA) was used for all statistical analyses in which the prognostic value of each miRNA was analyzed. The data are divided, at the value representing the median level of expression of a particular miRNA, into two groups with either high or low expression.

Other experimental materials and procedures are described in Additional file 2.

## Results

### Establishment and characterization of a new *in vitro* model of acquired resistance to letrozole

**Figure 1:**
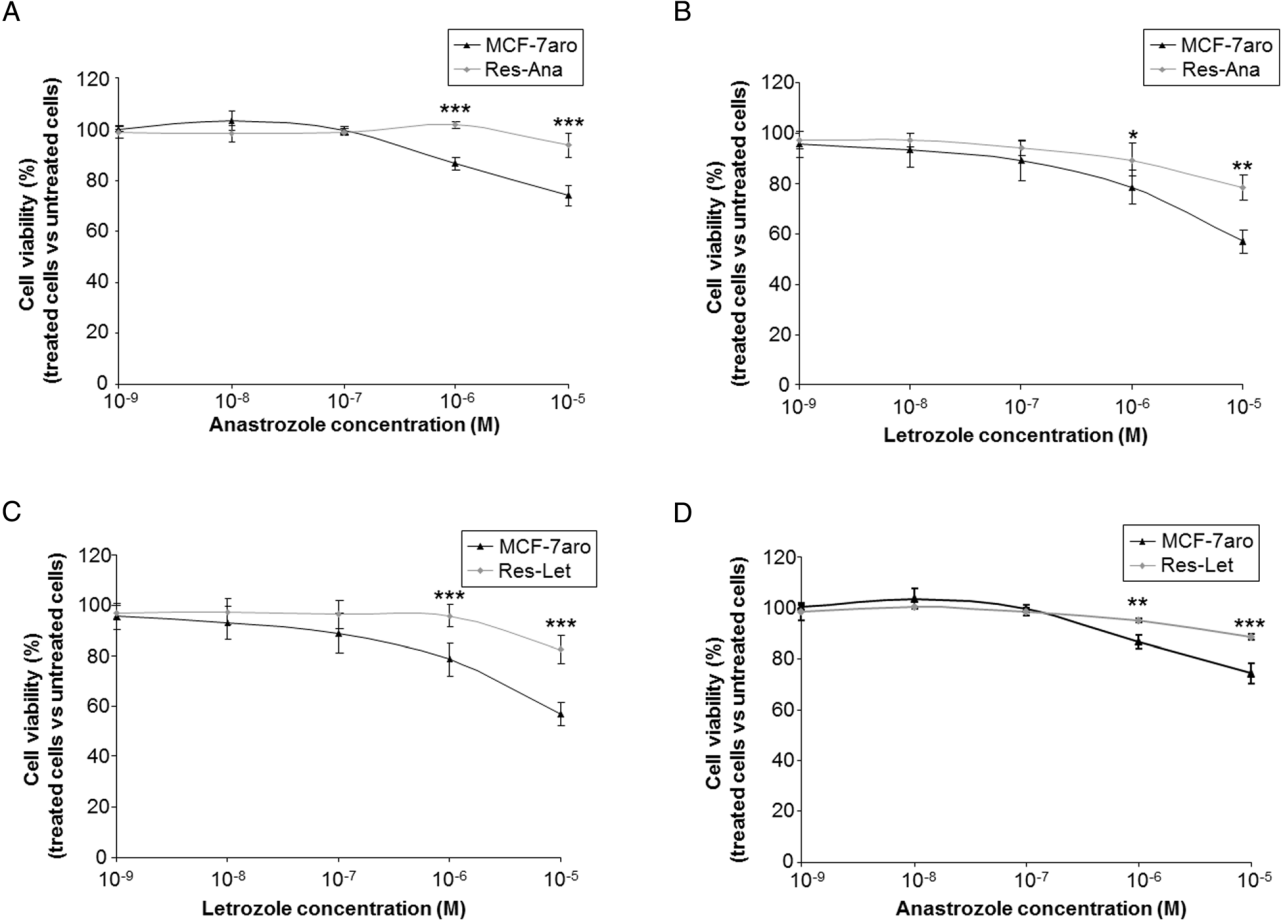
**Pharmacological response of resistant cell lines to aromatase inhibitors.** Pharmacological response of MCF-7aro and Res-Ana cells to anastrozole **(A)** or letrozole **(B)** and of MCF-7aro and Res-Let cells to letrozole **(C)** or anastrozole **(D)**, as assessed by cytotoxicity assays (mean ± SD from three independent experiments). *P < 0.05, **P < 0.01 and ***P < 0.001 versus the corresponding MCF-7aro (Student’s *t*-test).

Our group has previously described a model of acquired resistance to anastrozole (Res-Ana cells) [6] established by long-term exposure of MCF-7aro [28]. The resulting Res-Ana cells displayed significant resistance (in cytotoxicity assays) to anastrozole (Figure 1A), but they also decreased sensitivity to letrozole (Figure 1B). In this work we established (see the Methods section) and characterized a new cellular model of acquired resistance to letrozole, the Res-Let cells. The Res-Let cells displayed complete resistance to letrozole (Figure 1C) and, despite being selected under letrozole exposure only, also significantly decreased sensitivity to anastrozole, suggesting, at least in part, common mechanisms of resistance (Figure 1D).

As both ER-independent [2,10] and ER-dependent [39-41] mechanisms have been described in long-term AI resistance models, we investigated whether the AI resistance acquired by the Res-Let cells was due to any changes in aromatase function, ERα expression or ERα activity. We found no differences in aromatase activity and similar total inhibition between Res-Let and MCF-7aro cells in the presence of letrozole or anastrozole (Additional file 3: Figure S1A). Expression levels of ERα protein remained unchanged between the two cell lines, and we detected no phosphorylation at serine 118 or serine 167 in either cell line (Additional file 3: Figure S1B). Investigation of ERα transcriptional activity by estrogen response element-luciferase assay revealed that both the MCF-7aro and Res-Let cells displayed similar and low basal ERα activity levels (Additional file 3: Figure S1C), thus ruling out any ligand-independent activation of ERα (consistent with the absence of any phosphorylation at serines 118 and 167). 17β-estradiol (E_2_) treatment led to similar increased ERα activity and treatment with the selective estrogen receptor downregulator fulvestrant, which induces inhibition and degradation of ERα, abolished the E_2_-induced ERα activity (Additional file 3: Figure S1C). Altogether, and similarly to the Res-Ana cells [6], these data allowed us to rule out the acquired resistance of Res-Let cells being ascribed to an altered ERα expression or functionality, to an impaired activity of the aromatase, or to any loss of inhibitory effects of AIs on the aromatase.

### Identification of new deregulated miRNA signatures associated with acquired aromatase inhibitor resistance in Res-Let and Res-Ana cells

**Figure 2:**
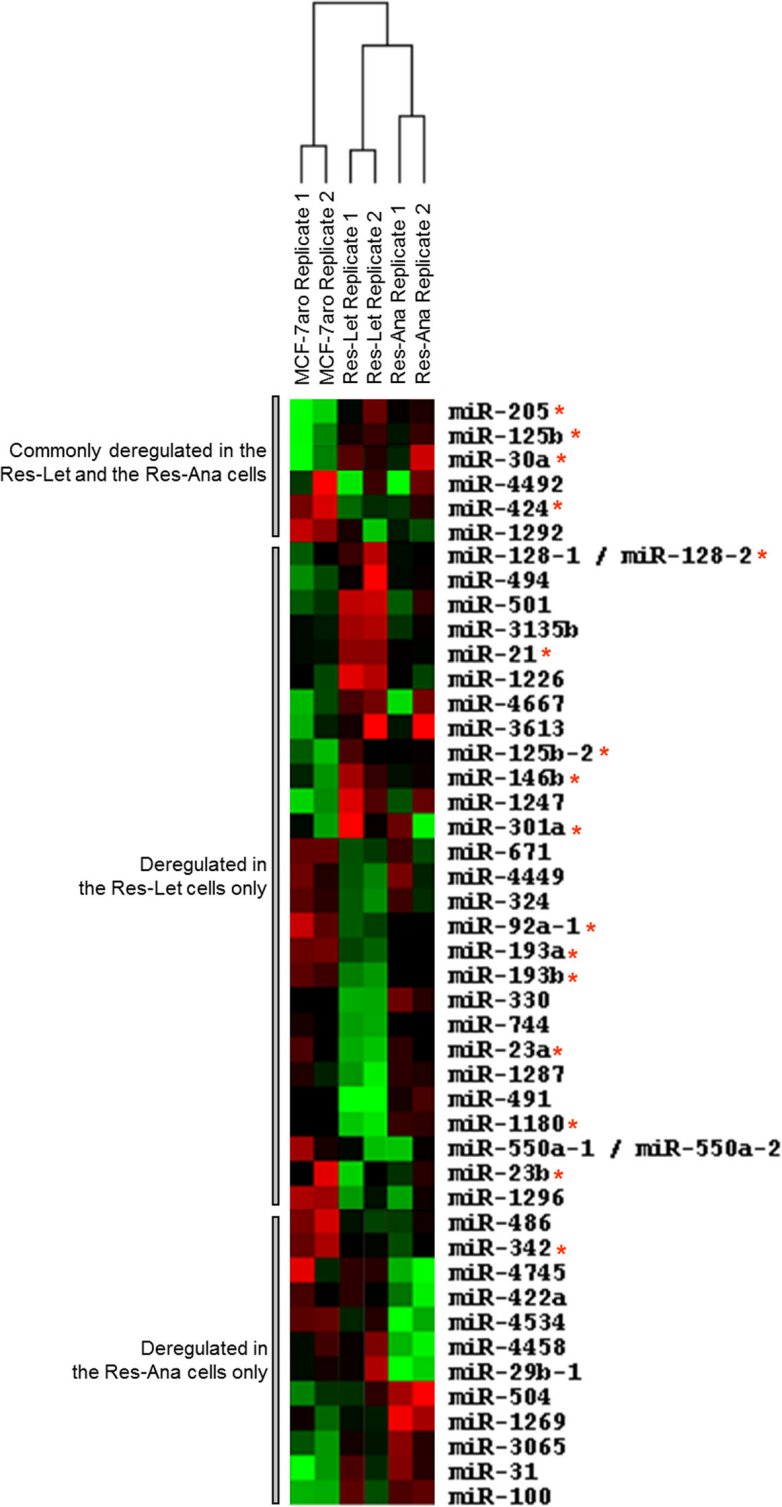
**Deregulated microRNA expression profiles associated with aromatase inhibitor resistance.** Dendogram of 45 micro RNA (miRNA) probe sets deregulated at the basal level in the Res-Let and Res-Ana cells as compared with the MCF-7aro cells in two independent cell cultures (replicates 1 and 2). Using the Cluster and TreeView software packages, the miRNAs were ordered according to their degree of similarity after hierarchical clustering of their expression profiles. Each column represents a cell line replicate, and each row represents a miRNA. Expression levels above the median are presented in red, and those below are shown in green. Red asterisks represent miRNAs previously described as associated with estrogen receptor (ER)/estrogen signaling, ER activity, breast cancer or endocrine resistance [16,42].

**Figure 3:**
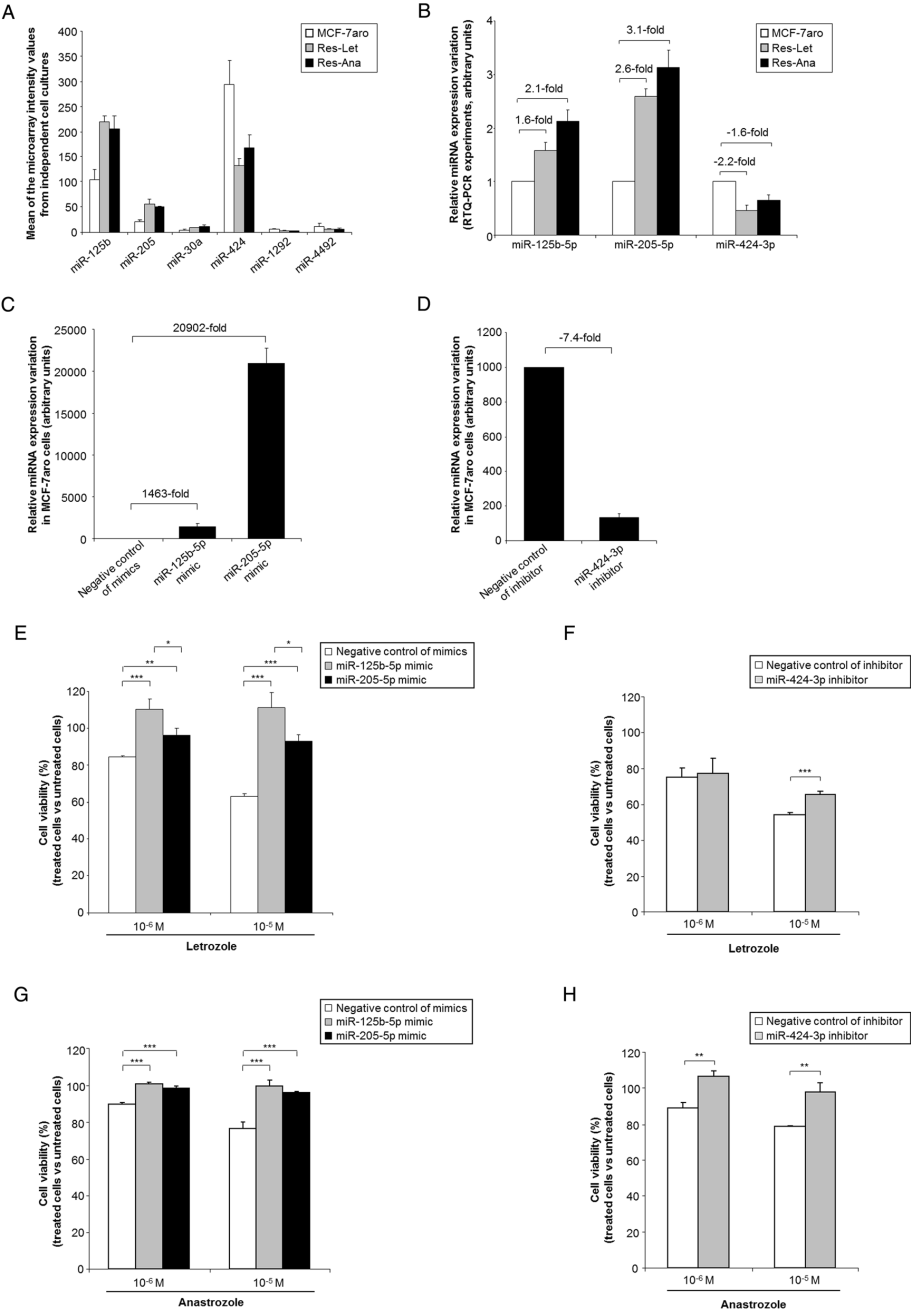
**MicroRNA-125b, microRNA-205 and microRNA-424 deregulations confer letrozole and anastrozole resistance. (A)** Microarray mean intensity values of the six microRNA (miR) probe sets commonly deregulated between Res-Let and Res-Ana cells. **(B)** Quantitative real-time PCR (RTQ-PCR) validation of miR-125b-5p-, miR-205-5p- and miR-424-3p-deregulated expression levels in Res-Let and Res-Ana cells versus MCF-7aro cells (mean ± SD from at least three independent experiments). **(C)** RTQ-PCR transfection efficiency validation in the MCF-7aro cells transfected with either the negative control of mimics, miR-125b-5p mimic, miR-205-5p mimic or **(D)** with either the negative control of inhibitor or miR-424-3p inhibitor (mean ± SD from at least three independent experiments). **(E)** Cell viability in response to letrozole in MCF-7aro cells transiently transfected with either the negative control of mimics, miR-125b-5p mimic, miR-205-5p mimic or **(F)** with the negative control of inhibitor or miR-424-3p inhibitor. **(G)** Cell viability in response to anastrozole in MCF-7aro cells transiently transfected with either the negative control of mimics, miR-125b-5p mimic, miR-205-5p mimic or (H) with either the negative control of inhibitor or miR-424-3p inhibitor. **(E)**, **(F)**, **(G)** and **(H)** are representative of at least three independent experiments. *P < 0.05, **P < 0.01 and ***P < 0.001 (Student’s *t*-test).

With the aim of selecting relevant deregulated miRNAs associated with acquired letrozole or anastrozole resistance, we analyzed miRNA expression profiles between MCF-7aro and AI-resistant cells from two independent cell culture replicates. The analysis led to the identification of miRNAs associated with AI resistance: 33 miRNAs reproducibly deregulated between the Res-Let and the MCF-7aro cells (15 upregulated and 18 downregulated), 18 miRNAs reproducibly deregulated (8 upregulated and 10 downregulated) between the Res-Ana and the MCF-7aro cells, of which 6 miRNAs similarly deregulated in both AI-resistant cell lines (Figure 2). Details of the corresponding FC values are presented in Table 1. Interestingly, 16 (approximately 36%) of these 45 miRNAs have previously been ascribed to “estrogen action” (that is, suppressed or stimulated under E_2_ or ERα ligands, regulated by ERα, regulating ERα, deregulated in breast cancer or deregulated in endocrine resistance) [16,42] (Figure 2, miRNAs with a red asterisk). In the only study in which miRNA expression profiles associated with AI resistance were investigated, upregulation of miR-128a (miR-128-1) was identified in letrozole-resistant cells [26]. Our data confirmed this (Figure 2 and Table 1), thus reinforcing the relevance of our AI resistance-associated miRNA signature. The finding of six miRNA array probe sets (miR-125b, miR-205, miR-30a, miR-424, miR-1292 and miR-4492) (Figure 3A) reproducibly and commonly deregulated in both the letrozole- and anastrozole-resistant cells suggests the existence of common molecular mechanisms associated with AI resistance. We selected for RTQ-PCR validation three candidate miRNAs (miR-125b-5p, miR-205-5p and miR-424-3p (corresponding respectively to the miR-125b, miR-205 and miR-424 array probe sets)), based on the following criteria: high expression values (Figure 3A) and belonging to the most deregulated miRNAs (Table 1). Validation by RTQ-PCR of the increased expression levels of miR-125b and miR-205 and decreased expression levels of miR-424 in both Res-Let cells and Res-Ana cells compared with MCF-7aro cells (Figure 3B) showed good consistency with the FC determined by microarray analysis (Table 1).

**Table 1:** **Deregulated microRNA expression associated with resistance to aromatase inhibitors**

**miRNA probe sets** ^**a**^	**Fold change** ^**b**^			
	**Res-Let**		**Res-Ana**	
	**Replicate 1**	**Replicate 2**	**Replicate 1**	**Replicate 2**
**miR-125b**	1.87	2.44	1.86	2.13
**miR-205**	2.76	2.86	2.19	2.99
**miR-30a**	1.71	3.17	2.97	2.15
**miR-424**	−2.33	−2.07	−1.76	−1.74
miR-1292	−3.02	−1.73	−2.06	−2.06
miR-4492	−2.51	−1.77	−2.14	−2.02
**miR-21**	1.73	1.70	NC	NC
**miR-125b-2**	1.70	1.79	1.94	NC
miR-128-1/miR-128-2	1.70	1.70	NC	NC
miR-146b	1.96	1.97	1.78	NC
**miR-301a**	1.80	2.63	NC	NC
**miR-494**	3.14	1.70	NC	NC
miR-501	2.28	2.46	NC	NC
miR-1226	2.28	1.98	NC	NC
miR-1247	2.09	3.85	2.20	NC
miR-3135b	1.98	1.81	NC	NC
miR-3613	2.68	1.93	2.76	NC
miR-4667	1.91	2.30	1.91	NC
miR-23a	−1.75	−2.24	NC	NC
**miR-23b**	−2.08	−1.98	−1.72	NC
**miR-92a-1**	−1.73	−2.55	NC	−1.86
**miR-193a**	−2.03	−1.82	NC	NC
miR-193b	−2.04	−2.07	NC	NC
miR-324	−1.88	−1.85	NC	NC
**miR-330**	−1.72	−1.72	NC	NC
miR-4449	−1.76	−1.94	NC	NC
miR-491	−2.63	−2.41	NC	NC
miR-550a-1/miR-550a-2	−2.05	−1.78	NC	−3.07
miR-671	−1.73	−1.85	−1.81	NC
miR-744	−1.78	−1.87	NC	NC
miR-1180	−2.14	−1.78	NC	NC
miR-1287	−1.80	−1.88	NC	NC
miR-1296	−1.83	−2.83	NC	−2.90
miR-31	NC	3.68	1.88	4.39
**miR-100**	NC	2.43	2.40	2.31
miR-504	NC	NC	2.70	2.61
miR-1269	NC	NC	2.41	2.10
miR-3065	NC	NC	1.86	2.05
**miR-29b-1**	NC	NC	−2.11	−2.22
**miR-342**	−1.77	NC	−1.74	−1.77
miR-422a	NC	NC	−2.21	−1.84
**miR-486**	−2.46	NC	−1.88	−1.80
miR-4458	NC	NC	−2.73	−1.75
miR-4534	NC	NC	−2.42	−3.51
miR-4745	NC	−1.80	−2.74	−3.74

^a^MicroRNAs (miRNAs) identified by the miRWalk database as being associated with a deregulated AKT pathway are shown in boldface type. ^b^Fold change (FC), corresponding to the ratio of the value for Res-Let or Res-Ana cells to that for MCF-7aro cells. Only probe sets with FC values superior to the cutoff selected (≥1.70 or less than or equal to −1.70) in the two independent cell culture replicates were considered to be differentially expressed. No change (NC) was defined as values −1.70 < FC <1.70.

### Deregulated expression of miR-125b, miR-205 and miR-424 confers *de novo* aromatase inhibitor resistance in MCF-7aro cells and aggressive features of endocrine resistance

We compared sensitive MCF-7aro cells transfected with either the mimic of miR-125b, the mimic of miR-205 or the inhibitor for miR-424 (Figures 3C and 3D) to their respective transfected negative controls, searching for any signs of acquisition of a phenotype similar to that developed by the AI-resistant cells. The MCF-7aro cells transfected with either miR-125b or miR-205 mimics became significantly resistant to letrozole (both at 10^−6^ M and 10^−5^ M), with a significantly greater impact of miR-125b (Figure 3E) that very closely resembled the resistance displayed by the Res-Let cells (Figure 1C). MiR-424 expression silencing under 10^−5^ M, but not 10^−6^ M, treatment conditions in MCF-7aro cells significantly decreased letrozole sensitivity (Figure 3F). As demonstrated in Figures 3G and 3H, ectopic overexpression of miR-125b or miR-205, or miR-424 silencing, all conferred to MCF-7aro cells complete resistance to both 10^−6^ M and 10^−5^ M anastrozole treatment, comparable to that displayed by the Res-Ana cells (Figure 1A). Altogether, these data demonstrate the successful *de novo* resistance to both letrozole and anastrozole achieved by mimicking in MCF-7aro cells the miR-125b, miR-205 or miR-424 expression pattern detected in the Res-Let and Res-Ana cells. Deregulated expression of miR-125b, miR-205 or miR-424 in ER+ breast cancer cells thus represents, at least in part, new molecular mechanisms involved in endocrine resistance to different AI drugs.

**Figure 4:**
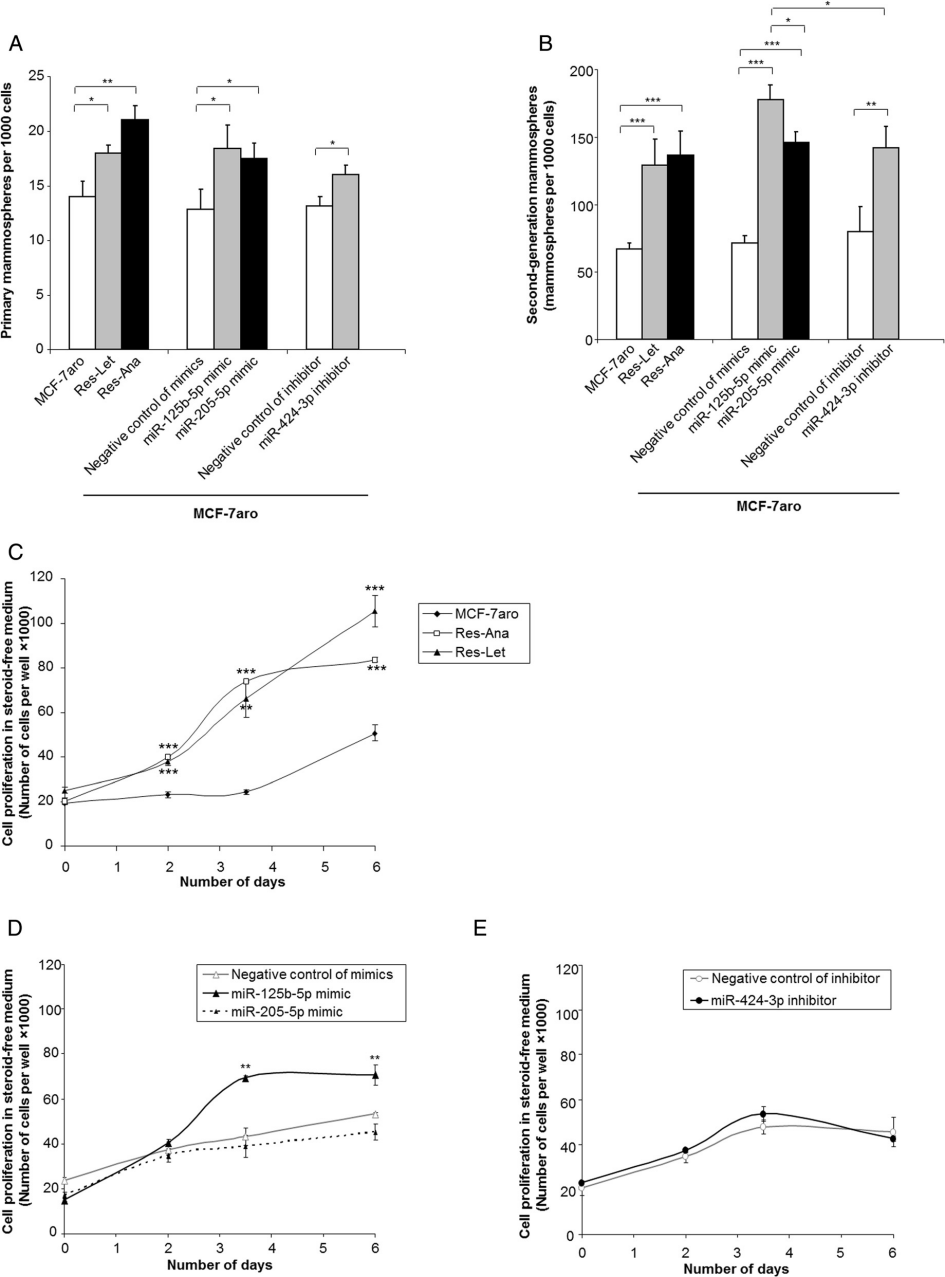
**MicroRNA-125b and microRNA-205 overexpression and microRNA-424 expression silencing increase aggressiveness in MCF-7aro cells. (A)** Primary mammospheres (PMs) formation assay. MCF-7aro, Res-Let, Res-Ana and MCF-7aro cells transfected by microRNA (miR)-125b-5p or miR-205-5p mimic, miR-424-3p inhibitor or their negative controls were cultured in nonadherent mammosphere culturing conditions. **(B)** Second-generation mammospheres generated from the PMs shown in **(A)**. Data given in **(A)** and **(B)** are the mean ± SD of three independent experiments. **(C)** Growth kinetics of MCF-7aro, Res-Ana and Res-Let cells in steroid-free medium. **(D)** Growth kinetics in steroid-free conditions of MCF-7aro cells transiently transfected with either the negative control of mimics, miR-125b-5p or miR-205-5p or **(E)** the negative control of inhibitor or miR-424-3p inhibitor. **(C)**, **(D)** and **(E)** show the results representative of at least three independent experiments. *P < 0.05, **P < 0.01 and ***P < 0.001 (Student’s *t*-test).

A growing body of evidence supports the presence within the tumor of heterogeneous subpopulations of cells, including cancer stem cells or tumor-initiating cells (TICs) with self-renewing properties generally associated with an aggressive phenotype, escape from chemotherapy or endocrine therapy, and the promotion of metastases [6,43,44]. Indeed, residual breast cancer cells after letrozole therapy have been shown to display TIC features [44], and, compared with MCF-7aro cells, a higher percentage of TICs with self-renewing properties have been detected in letrozole- or anastrozole-resistant cells [6,45]. We investigated self-renewal cell capacity by assessing the efficiency of these cells in generating nonadherent mammospheres (mammosphere-forming efficiency (MFE)). As shown in Figure 4A, nonadherent mammosphere formation was significantly enriched starting from Res-Let or Res-Ana cell populations compared with that from the MCF-7aro cell population. Strikingly, transfection of MCF-7aro cells with the miR-125b or miR-205 mimics or with the miR-424 inhibitor increased MFE as compared with their respective negative control (Figure 4A). We then tested the self-renewal capability of the mammosphere-forming cells by the PMs dissociated into single cells and performed second-generation mammosphere assays. The proportion of mammosphere-forming cells isolated from the PMs greatly increased through the second passage, thus indicating their ability to self-renew. MiR-125b mimic transfection had the greatest MFE-increasing impact (Figure 4B).

Several studies have now established that endocrine resistance in ER+ breast cancer cells often involves a switch from steroid signaling and endocrine therapy responsiveness to an estrogen-independent phenotype, and in particular estrogen-independent growth properties [46]. Consistently with these observations, both the Res-Let and Res-Ana cells displayed a significantly greater magnitude of proliferation compared with MCF-7aro control cells in estrogen-deprived conditions (Figure 4C), highlighting their acquired increased capacity to proliferate in the absence of any ERα agonist. Strikingly, ectopic overexpression of miR-125b in MCF-7aro cells induced estrogen-independent growth in steroid-free conditions (Figure 4D). Conversely, ectopic overexpression of miR-205 or miR-424 expression silencing had no impact (Figures 4D and 4E).

Altogether, these data show that mimicking the deregulated expression of three candidate miRNAs observed in two cellular models of acquired AI resistance imposes on the sensitive MCF-7aro cells: (1) a *de novo* pharmacological resistance to both anastrozole and letrozole, (2) a selection of stem-like and tumor-initiating cells possessing cell renewing properties and (3) for miR-125b only, estrogen-independent growth properties.

### Deregulated expression levels of miR-125b, miR-205 or miR-424 are sufficient to activate the PI3K/AKT/mTOR pathway

Target prediction analysis (DIANA-mirPath analysis) of the miRNAs found to be deregulated in the Res-Let cells allowed the identification of several significantly deregulated Kyoto Encyclopedia of Genes and Genomes (KEGG) signaling pathways (Table 2) (*P*-values from 10^−9^ to 10^−3^). Interestingly, 7 of the 13 top-ranked signaling pathways identified converged on the AKT pathway (phosphatidylinositol signaling system, mTOR signaling pathway, focal adhesion, insulin signaling pathway, ErbB signaling pathway, MAPK signaling pathway, tight junction). Similarly, when we focused on the Res-Ana miRNA signature, we found that 5 of the 11 top-ranked KEGG signaling pathways identified after DIANA-mirPath analysis also converged on the AKT pathway and were identified by the Res-Let miRNA signature (Table 2) (*P*-value*s* from 10^−10^ to 10^−4^).

**Table 2:** **The top-ranked signaling pathways associated with resistance to aromatase inhibitors** ^**a**^

		**Deregulation in Res-Let vs MCF-7aro**	**Deregulation in Res-Ana vs MCF-7aro**	**Convergence on the AKT pathway**
**KEGG pathway**	**Pathway ID**	***P*** **-value** ^**b**^	***P*** **-value** ^**b**^	
Wnt signaling pathway	hsa04310	7.1 × 10^−9^	7.0 × 10^−7^	
TGF-β signaling pathway	hsa04350	2.1 × 10^−8^	3.0 × 10^−4^	
Axon guidance	hsa04360	2.9 × 10^−8^	7.2 × 10^−8^	
Regulation of actin cytoskeleton	hsa04810	1.0 × 10^−7^	1.5 × 10^−5^	
MAPK signaling pathway	hsa04010	1.3 × 10^−7^	1.8 × 10^−4^	Yes
Focal adhesion	hsa04510	3.0 × 10^−7^	8.2 × 10^−10^	Yes
Adherens junction	hsa04520	2.0 × 10^−6^	2.4 × 10^−6^	
Insulin signaling pathway	hsa04910	1.6 × 10^−5^	3.5 × 10^−10^	Yes
Ubiquitin-mediated proteolysis	hsa04120	1.1 × 10^−4^	4.5 × 10^−5^	
ErbB signaling pathway	hsa04012	1.3 × 10^−4^	4.5 × 10^−6^	Yes
Tight junction	hsa04530	7.1 × 10^−4^	NP	Yes
Phosphatidylinositol signaling system	hsa04070	1.3 × 10^−3^	NP	Yes
mTOR signaling pathway	hsa04150	3.0 × 10^−3^	2.8 × 10^−5^	Yes

^a^MAPK, Mitogen-activated protein kinase; mTOR, Mammalian target of rapamycin; NP, Not present in the top-ranked signaling pathways; TGF-β, Transforming growth factor β. ^b^ *P*-value was obtained using the DIANA-mirPath algorithm to identify significantly deregulated Kyoto Encyclopedia of Genes and Genomes (KEGG) signaling pathways predicted to be altered by the microRNAs selected in our study (Table 1).

**Figure 5:**
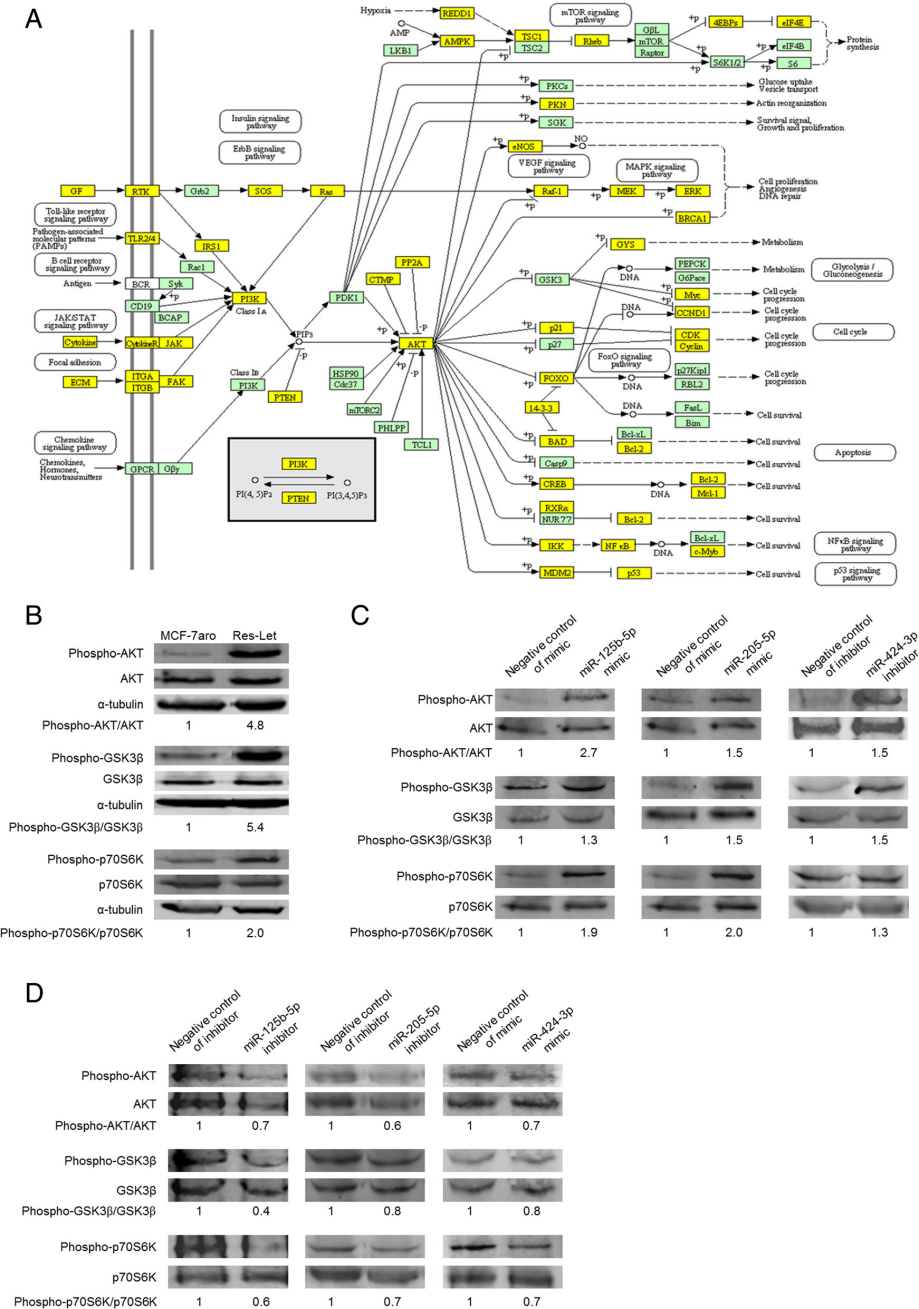
**The PI3K/AKT/mTOR pathway is targeted by the microRNAs deregulated in the aromatase inhibitor-resistant cells. (A)** Overview of the AKT signaling pathway illustrating the results of our analysis. Genes in yellow rectangles represent the genes targeted by microRNAs (miRs) miR-125b, miR-205, miR-424 and miR-30a. **(B)** Western blot analysis of the AKT pathway in MCF-7aro and Res-Let cells. **(C)** Western blot analysis of the AKT pathway in MCF-7aro cells transfected with the mimic of miR-125b-5p or miR-205-5p, the inhibitor of miR-424-3p, or their respective negative controls. **(D)** Western blot analysis of the AKT pathway activation status in Res-Let cells transfected with the inhibitor of miR-125b-5p or miR-205-5p, the mimic of miR-424-3p, or their respective negative controls. **(B)**, **(C)** and **(D)** show representative blots from three independent experiments and cell lysates. The relative expression levels of the corresponding ratio of phosphorylated to total proteins were quantified, and these data are shown below the blots.

A query using the miRWalk database revealed that 16 miRNAs (approximately 36%) are described as targeting the AKT pathway (miRNAs in boldface type in Table 1). Of the six miRNAs commonly deregulated in Res-Let and Res-Ana cells, only four could be analyzed by DIANA-mirPath (miR-125b, miR-205, miR-424 and miR-30a), which revealed that all four were sufficient to identify the AKT pathway. In addition, the information captured by both the DIANA-mirPath and miRWalk database analyses revealed that 29% (98 of 338) of the genes belonging to the AKT pathway are candidate targets for these four miRNAs (Figure 5A).

Validating these *in silico* predictions, the Res-Let cells displayed a constitutive activation of the AKT/mTOR pathway, as revealed by the activation of AKT (increase in the phospho-AKT/total AKT ratio), coupled with an increase in the phosphorylation level of the AKT downstream target GSK3β and an increase in the phosphorylation level of the mTOR downstream target p70S6K (Figure 5B). Such constitutive activation of the AKT/mTOR pathway is also present in the Res-Ana cells as compared with the MCF-7aro cells and has previously been described by our group [6]. Strikingly, transfecting MCF-7aro cells with either the mimic of miR-125b, the mimic of miR-205 or the inhibitor for miR-424 was sufficient to confer activation of the AKT/mTOR pathway (Figure 5C). Conversely, transfecting Res-Let cells with either the inhibitor of miR-125b, the inhibitor of miR-205 or the mimic of miR-424 led to a markedly decreased activation of the AKT/mTOR pathway (Figure 5D and Additional file 4: Figure S2). The AKT/mTOR survival pathway plays a pivotal role in endocrine resistance, and its activation is sufficient to confer *de novo* resistance to anastrozole [6] or to letrozole (Additional file 5: Figure S3 and [47]). Altogether, these findings provide new evidence favoring the development by AI-resistant cells of such a mechanism of deregulated expression of specific miRNAs that leads to the constitutive activation of the AKT/mTOR pathway.

### MiR-125b expression levels are associated with poor prognosis and with relapse in ER+ breast cancer patients treated with endocrine therapy

**Figure 6:**
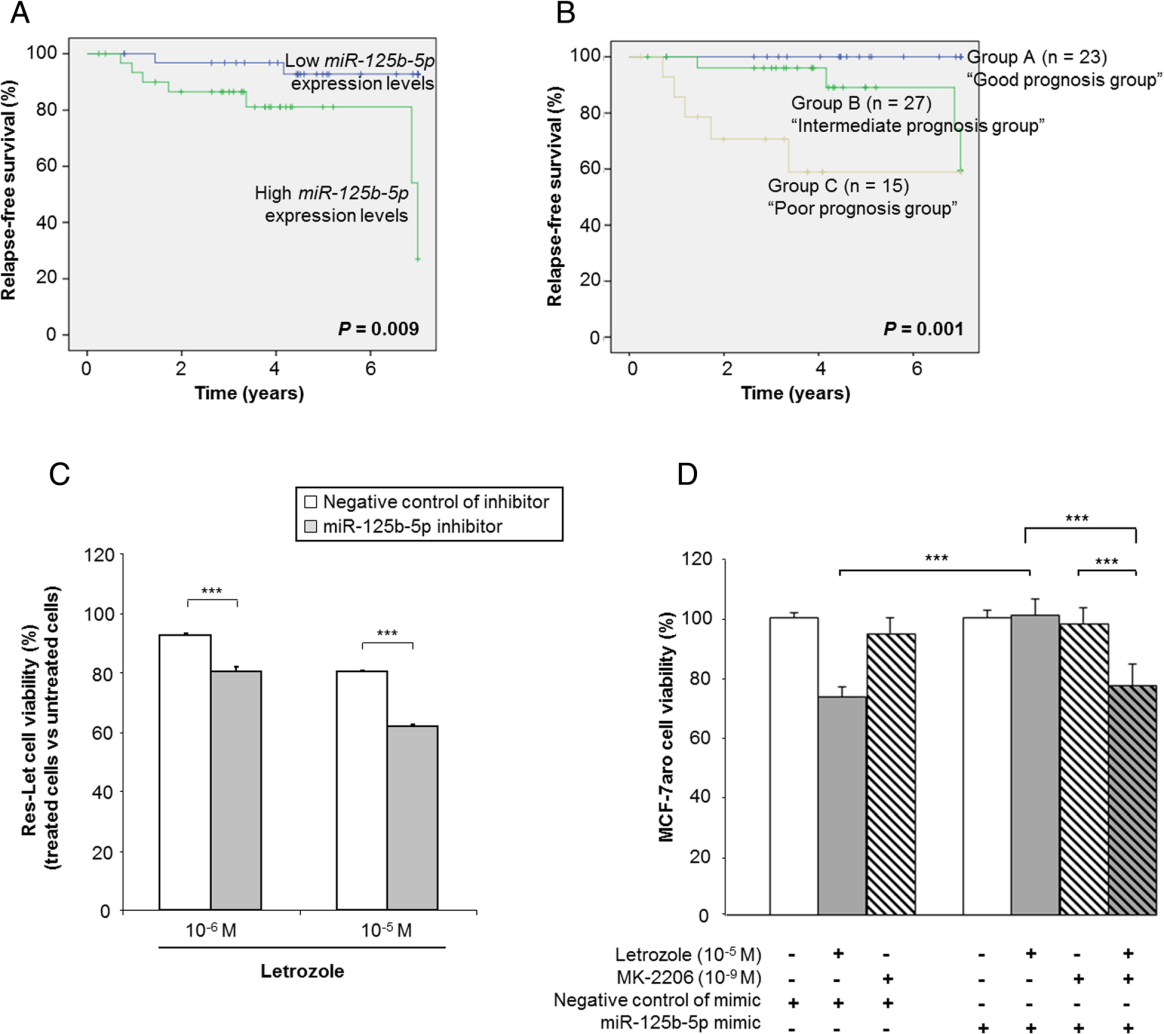
**MicroRNA-125b is a poor prognostic biomarker and represents a therapeutic target to overcome letrozole resistance. (A)** Kaplan-Meier analysis (log-rank test) for relapse-free survival (RFS) is shown in a cohort of primary breast tumor samples (*n* = 65). **(B)** Effect of the microRNA (miR) miR-125b-5p expression level and lymph node status signature on RFS among our cohort with 65 breast tumors. **(C)** Cell viability in response to letrozole in Res-Let cells transiently transfected with either miR-125b-5p inhibitor or its negative control. **(D)** Cell viability in response to letrozole (10^−5^ M) and/or MK-2206 (10^−9^ M) in MCF-7aro cells transiently transfected with either miR-125b-5p mimic or its negative control. **(C)** and **(D)** are representative of at least three independent experiments. ***P < 0.001 (Student’s *t*-test).

To investigate the clinical relevance of miR-125b, miR-205 and miR-424, we used RTQ-PCR to explore the expression levels of these three miRNAs in a cohort of 65 primary breast tumor samples (Additional file 1: Table S1). Strikingly, high expression levels of miR-125b, though not miR-205 or miR-424, were associated with shorter RFS (*P* = 0.009 by log-rank test) (Table 3 and Figure 6A). The prognostic value of miR-125b expression levels was more informative than the SBR grade (log-rank test) (Table 3), and the results of Fisher’s exact test analysis revealed that miR-125b expression levels were not associated with SBR grade (Additional file 6: Table S2). The prognostic value of miR-125b expression levels was also more informative than the ER, PR or HER2 conventional biomarkers in this cohort (log-rank test) (Table 3). Lymph node status was associated with shorter RFS (*P* = 0.006 by log-rank test) (Table 3). Prognostic factors for RFS with a 0.05 significance level in univariate analysis were then tested into a multivariate Cox model using a backward selection procedure. Both miR-125b levels and lymph node status persisted in the model (*P* < 0.05), revealing that these biomarkers are independent prognostic markers (Table 3).

**Table 3:** **Univariate and multivariate analyses of the prognostic value of miR-125b-5p, miR-205-5p and miR-424-3p expression levels and clinical parameters in relation to relapse-free survival** ^**a**^

	**Number of samples**	**Univariate**		**Multivariate**	
		**HR (95% CI)**	***P*** **-value** ^**b**^	**HR (95% CI)**	***P*** **-value** ^**b**^
miR-125b-5p expression levels	65	6.80 (1.35 to 35.26)	0.009	6.35 (1.21 to 33.27)	0.029
miR-205-5p expression levels	65	1.86 (0.63 to 10.40)	NS (0.17)	ND	ND
miR-424-3p expression levels	65	1.04 (0.52 to 7.58)	NS (0.31)	ND	ND
Age (≤50 yr; >50 yr)	65	0.02 (0.54 to 2.03)	NS (0.90)	ND	ND
Macroscopic tumor size (≤30 mm; >30 mm)	64^c^	1.47 (0.08 to 1.89)	NS (0.22)	ND	ND
Lymph node status (≤3; >3)	65	7.68 (1.42 to 33.15)	0.006	6.29 (1.27 to 31.07)	0.024
Histological grade (SBR1 + SBR2; SBR3)	65	0.45 (0.35 to 8.18)	NS (0.50)	ND	ND
Histological grade (SBR1; SBR2)	22	1.41 (0.01 to 3.49)	NS (0.23)	ND	ND
Histological grade (SBR1; SBR3)	47	0.48 (0.06 to 4.00)	NS (0.49)	ND	ND
Histological grade (SBR2; SBR3)	61	1.10 (0.36 to 23.68)	NS (0.29)	ND	ND
ER status (negative; positive)	65	0.17 (0.20 to 2.85)	NS (0.68)	ND	ND
PR status (negative; positive)	65	0.10 (0.21 to 3.04)	NS (0.75)	ND	ND
HER2 status (negative; positive)	65	0.78 (0.48 to 6.84)	NS (0.38)	ND	ND

^a^95% CI, 95% confidence interval; ER, Estrogen receptor; HER2, Human epidermal growth factor receptor 2; HR, Hazard ratio; miR, MicroRNA; ND, Not done; NS, Not significant; PR, Progesterone receptor; SBR, Scarff-Bloom-Richardson grade. ^b^ *P* < 0.05 was considered significant. ^c^Information available for 64 patients.

We then constructed a signature based on miR-125b expression levels and lymph node status by dividing patients into three groups: patients expressing low levels of miR-125b and having no or three or fewer involved lymph nodes (group A), patients expressing high levels of miR-125b and no or three or fewer involved lymph nodes or those having more than three involved lymph nodes and low expression of miR-125b (group B) and patients expressing high levels of miR-125b and more than three involved lymph nodes (group C). The resulting Kaplan-Meier curves for RFS, shown in Figure 6B (*P* = 0.001), indicated the stratification of the cohort into a “good prognosis group” (group A), an “intermediate prognosis group” (group B) and a “poor prognosis group” (group C). Finally, we tested the miR-125b expression levels and lymph node status signature with respect to RFS and found that the model associating miR-125b expression levels and lymph node status was a better fit (likelihood = 51.26) than either that with miR-125b expression levels only (likelihood = 59.05, *P* = 0.005) or that with lymph node status only (likelihood = 58.51, *P* = 0.007). This demonstrated that, in this cohort, the miR-125b expression level and lymph node status signature has the best prognostic value.

We then performed univariate analysis of the prognostic value of miR-125b expression levels with regard to RFS in the HR+/HER2− breast cancer subclass, and found that high expression levels of miR-125b were significantly associated with shorter RFS (*P* = 0.03 by log-rank test) (Table 4). Conversely, miR-205 or miR-424 expression levels displayed no prognostic value in the HR+/HER2− breast cancer subclass (*P* = 0.5 and *P* = 0.8, respectively, by log-rank test) (data not shown). Interestingly, high levels of miR-125b expression were still significantly associated with shorter RFS in luminal A breast cancers (*P* = 0.05) (Table 4), but they were not informative in luminal B breast cancers (Table 4). Altogether, these data suggest that miR-125b expression would add significant prognostic value to the luminal A subtype, where miR-125b expression would differentiate between excellent and intermediate or poor luminal A relapse-free survivors.

**Table 4:** **Univariate analysis of the prognostic value of miR-125b-5p expression levels in relation to relapse-free survival in different hormone receptor–positive breast cancers** ^**a**^

	**Number of samples**	**Univariate analysis**		
		**Hazard ratio**	**95% CI**	***P*** **-value** ^**b**^
All breast tumor samples	65	6.80	1.35 to 35.26	0.009
HR+/HER2− subclass	30	4.81	N/A	0.03
Luminal A subclass (HR+, HER2−, SBR1 or SBR2)^c^	19	3.75	N/A	0.05
Luminal B subclass (HR+, HER2+ and/or SBR3)^c^	17	1.69	0.07 to 17.12	0.96

^a^95% CI, 95% confidence interval; HER2, Human epidermal growth factor receptor 2; HR+, Hormone receptor–positive (estrogen receptor (ER) and/or progesterone receptor (PR)); miR, MicroRNA; N/A, Not applicable as there is no relapse event in the low miR-125b-5p expression level group; SBR, Scarff-Bloom-Richardson grade. ^b^ *P* < 0.05 was considered significant. ^c^Subclasses of breast cancer were determined using immunohistological (ER, PR, HER2) and SBR grades according to the St Gallen recommendation [38].

We aimed to investigate the association between the miR-125b marker and the clinical response of the HR+ breast cancer patients treated with endocrine therapy alone or in combination with chemotherapy. In the HR+/HER2− and luminal A subclasses, all patients except one were treated with endocrine therapy alone or in combination with chemotherapy; removing this patient from the HR+/HER2− or the luminal A subclass had no impact on the statistical significance of the results in univariate analysis (*P* = 0.03 and *P* = 0.05, respectively) (data not shown). We used Fisher’s exact test to investigate the association between the miR-125b marker and the clinical response of all the HR+ breast cancer patients treated with endocrine therapy alone or in combination with chemotherapy, whose clinical response was fully known at the 7-year follow-up examination. Whereas miR-205 and miR-424 expression levels showed no significant association, high miR-125b expression levels were associated with earlier relapse in this HR+ subgroup (*P* = 0.02) (Additional file 7: Table S3). Only four patients were treated with endocrine therapy alone, one of whom (treated with letrozole) displayed high miR-125b expression levels in the tumor sample and relapsed (and died at 1.7 years). The other three patients had not relapsed at the 7-year follow-up examination. Altogether, these data suggest that miR-125b expression levels are a novel biomarker of poor prognosis in breast cancer and are associated with earlier relapse in HR+ breast cancer treated with endocrine therapy.

### Targeting miR-125b- or miR-125b-driven AKT activation increases sensitivity to letrozole and overcomes resistance

Because high expression levels of miR-125b led to AI resistance and activation of the AKT/mTOR pathway in breast cancer cells and were also associated with poor prognosis among our cohort, we hypothesized that acquisition of deregulated miR-125b expression represents an alternative trick used by HR+ AI-resistant breast cancer cells to activate this crucial survival pathway. Interestingly, in contrast to the Res-Let and Res-Ana cells, new cellular models of acquired resistance to 4-hydroxy-tamoxifen (OH-Tam) (Res-Tam cells) or to fulvestrant (Res-Fulv cells), established by long-term exposure of MCF-7aro to the corresponding drugs (Additional file 8: Figures S4A and S4B), displayed no clear constitutive activation of the AKT/mTOR pathway (Additional file 8: Figure S4C). This suggests that these specific Res-Tam and Res-Fulv cells have developed alternative mechanisms of endocrine resistance. Interestingly, neither of these two cell lines displayed any deregulated miR-125b expression levels (Additional file 8: Figure S4D). Altogether, these data suggest that increased miR-125b expression levels in the Res-Let and Res-Ana cells, as compared with control cells, constitute an important mechanism developed by these two AI-resistant cells that leads to activation of the AKT/mTOR pathway.

We then wished to determine whether miR-125b and AKT activation paired with miR-125b overexpression would constitute relevant targets to overcome letrozole resistance. Strikingly, silencing miR-125b in Res-Let cells, paired with a decreased activation of the AKT/mTOR pathway (Figure 5D), led to a significant increase in sensitivity to letrozole (Figure 6C). We also investigated the impact of a pan-AKT inhibitor, MK-2206, on the letrozole response of MCF-7aro cells transfected by the mimic of miR-125b. MK-2206 is a highly selective non-ATP–competitive, allosteric AKT inhibitor that inhibits the activities of the three human AKT isoforms. Our group has previously verified that exposure to MK-2206 induces inhibition of the AKT pathway (significant decrease in the phosphorylation status of AKT, together with a significant decrease in the phosphorylation levels of GSK-3β and p70S6K [6]). Strikingly, in the MCF-7aro cells transfected by the mimic of miR-125b, combining the pan-AKT inhibitor MK-2206 with letrozole significantly restored sensitivity to letrozole, as shown by the significant decrease in cell viability compared with MK-2206 or letrozole alone (*P* < 0.001) (Figure 6D). More interestingly, the restored sensitivity to letrozole became similar to the letrozole sensitivity observed in MCF-7aro cells transfected by the negative control mimic (Figure 6D). Altogether, these data suggest that, in addition to its likely being a marker of poor prognosis in breast cancer cells, miR-125b may also represent a candidate therapeutic target to counteract letrozole resistance.

## Discussion

Although AI endocrine therapy provides obvious clinical benefits, the molecular mechanisms involved in resistance to AIs remain poorly described. To gain further insight into the molecular mechanisms underlying the AI resistance, recent whole-genome analyses using AI pretreatment tumor biopsies accrued from patients in AI neoadjuvant studies to delineate the mutational landscape associated with AI response strategies are of great interest [48]. However, for preclinical studies, few cellular models mimicking resistance to AIs are currently available. Because breast cancer cell lines usually display very low or no expression of endogenous aromatase [28], the most relevant cells in which to study the response to AIs are cells transfected with the human aromatase gene. Among the cellular models proposed to study AI resistance, aromatase-transfected and long-term estrogen-deprived (LTED) cell lines have been proposed, based on the hypothesis that lack of hormone in the environment could mimic the withdrawal of estrogen that occurs during treatment with AIs [39,49]. Most preclinical studies investigating AI resistance have been conducted on LTED models, but it has previously been shown that LTED cells are not totally equivalent to models of endocrine therapy-acquired resistance [26,39,49,50]. Indeed, genome-wide analysis revealed important gene expression profile differences between the AI-resistant and LTED cell lines and also among the AI-resistant cell lines themselves [39]. Supporting data came from the observation that activated signaling pathways observed in LTED cells were different from those observed in long-term AI-treated cells [49,50]. More importantly, the only study in which miRNA expression profiles associated with AI resistance were investigated clearly demonstrated that specific miRNA profiles could be inversely regulated between AI-resistant cells and LTED cells [26]. In particular, miR-128a, which was found to regulate TGF-β signaling in letrozole-resistant breast cancer cells, was upregulated in letrozole-resistant cells, but not in LTED cells [26]. Thus, cellular models established according to a protocol that mimics clinical treatment [2,6,39,50,51] (that is, long-term exposure to AIs) may more closely reflect the clinical situation and may be pertinent to investigation of acquired AI resistance. Few of such *in vitro* models are currently available [2,5,6,39,41].

Deciphering miRNA deregulations as new mechanisms associated with acquired AI endocrine resistance remains poorly investigated, and to date only one previous study team, using an “omics miRNA” approach, identified one particular miRNA associated with letrozole resistance [26]. In the present study, we aimed to capture a global view of the miRNA expression profiles associated with letrozole and anastrozole resistance in the hope of identifying common miRNA-targeted specific functional networks and relevant miRNAs (candidate biomarkers or putative therapeutic targets of ER+ breast cancers treated by AIs). We performed a large-scale miRNA analysis using two *in vitro* cellular models of acquired AI resistance (Res-Let and Res-Ana cells). Importantly, these cells are total populations of AI-resistant cells and may thus also mimic the heterogeneous phenotype and behavior of resistant cells possibly present in the tumors of patients who relapse under AI endocrine therapy.

The AKT/mTOR pathway is known to play a pivotal role in AI resistance [2,5,6,39,41]. Among the molecular mechanisms leading to activation of the AKT/mTOR pathway in AI-resistant cells, special attention has previously been given to the upstream ErbB family receptors [52]. Importantly, in a recent study, researchers found that in breast cancer cells with acquired letrozole resistance, the constitutive activation of the AKT/mTOR pathway could be blocked by PI3K and/or mTOR inhibitors, but not by EGFR/ErbB2 inhibitors [2]. This observation strongly supports the existence of a mechanism other than activation of receptors upstream of the AKT pathway. Our data show, for the first time to our knowledge, that acquisition of deregulated miRNA expression represents an alternative trick used by ER+ AI-resistant breast cancer cells to activate this crucial survival pathway and thus escape from AI endocrine therapy.

A growing body of evidence developed over the last several years supports the existence, within a given tumor, of separate cell subpopulations that have acquired different mechanisms, all converging to activate the same functional survival network [53]. Consistent with this idea, the Res-Let or Res-Ana cells develop a deregulated network of several miRNAs, each independently capable of leading (or predicted to lead) directly or indirectly to the activation of the AKT/mTOR pathway. Among the miRNAs identified in our study, deregulated expression of miR-23b [54], miR-494 [55], miR-21 [56-58], miR-301 [59] or miR-193a [60] has previously been shown to induce the activation of the AKT pathway in different cancer contexts. Among the miRNAs predicted to target the AKT/mTOR pathway and commonly deregulated in both Res-Let and Res-Ana cells, we then investigated miR-125b, miR-205 and miR-424 further. Our study has shown, for the first time to our knowledge, that ectopic overexpression of either miR-125b or miR-205, or silencing miR-424 expression, in the sensitive ER+ MCF-7aro cells was sufficient to trigger activation of the AKT/mTOR pathway, to develop *de novo* resistance to both letrozole and anastrozole drugs and to induce the selection of stem-like and tumor-initiating cells possessing self-renewing properties.

Several previous studies have demonstrated that any given miRNA could play a dual role (via oncogenic or tumor-suppressive activities), depending on the tissue or the tumor. This is likely due to any given miRNA targeting multiple mRNAs, each of which has a different function in an individual cellular context [11-13,61]. Consistently, miR-424 is known to play a suppressive role in some tumors [62], whereas high expression levels have been ascribed to chemotherapy resistance [63,64]. In accordance with our results, decreased levels of miR-424 led to an activated AKT/mTOR pathway in prostate and colon cancer cells [65]. MiR-205 also plays a dual role in carcinogenesis [13]. In its “devil” role (associated with tumorigenesis), and in accordance with our study, overexpression of miR-205 has been shown to lead to a coordinated activation of the AKT signaling pathway in several cancer contexts [66-69]; to our knowledge, though, it has never been shown in breast cancer cells. Complexity is further revealed by the observation that, conversely, miR-205 is able to inhibit activation of the AKT pathway [70]. However, it is noteworthy that this latter study was conducted in ERα-negative breast cancer cells, and one can suggest that the ER status of breast cancer cells provides a cellular context whereby miR-205 exerts opposite roles.

MiR-125b interferes with many different cellular processes and, according to the cellular context, acts as a tumor suppressor (for example, gliomas, ovarian cancer, and hepatocellular carcinoma), as an oncogene (for example, non-small cell lung carcinoma, colon cancer) or as both (brain tumors or prostate cancer) [12,61]. In its tumor-suppressive functions, miR-125b has been reported to target *ENPEP*, *CK2*-α, *CCNJ*, *MEGF9* [71] or the proto-oncogene *ETS1* [72]. Conversely, in its “devil” role, high miR-125b expression levels confer to breast cancer cells resistance to paclitaxel by targeting *BAK1* [73] and are detected in miRNA signatures present in Tam- or fulvestrant-resistant breast cancer cell lines [17,18]. In a neoadjuvant chemotherapy study in breast cancer, high expression levels of circulating miR-125b were detected in nonresponsive patients [74]. One of the key findings of our study is that miR-125b overexpression not only enables activation of the AKT/mTOR pathway and AI resistance but also confers an estrogen-independent phenotype to the sensitive ER+ MCF-7aro cells. Importantly, miR-125b is known to directly target the p53 tumor suppressor gene and other genes belonging to the p53 network [12,61], and p53 is known to closely interfere and communicate with the cancer relevant PI3K/AKT/mTOR pathway [75-78]. Indeed, p53 is able to regulate cell survival by inhibiting the PI3K/AKT/mTOR survival pathway, such as by positively regulating the expression of AKT/mTOR pathway inhibitors [76,78] or by negative regulating the expression of *PI3KCA*, encoding the p110α catalytic subunit of PI3K [75,77]. Thus, inactivation of p53 and subsequent upregulation of *PI3KCA* is one of the mechanisms contributing to the pathophysiology of cancer [75,77]. Interestingly, transfecting MCF-7aro cells with the mimic of miR-125b was sufficient to lead to decreased p53 expression levels (at both the mRNA and protein levels) and to subsequent increased p110α expression levels (Additional file 9: Figure S5). These data strongly support that p53-driven mechanisms could be involved in the activation of the AKT pathway in the presence of miR-125b. This could thus represent one possible miR-125b-driven mechanism developed by the AI-resistant cells.

Our work also highlights, for the first time to our knowledge, the clinical relevance of miR-125b in breast cancer by demonstrating that high expression levels of this miRNA may represent a marker of poor prognosis. Indeed, assessing miR-125b expression levels (alone or associated with lymph node status) may allow the restratification of patients with breast cancer into outcome-dependent subclasses. Most importantly, high miR-125b expression levels were also associated with earlier relapse in ER+ breast cancer treated with endocrine therapy. The measurement of miRNA expression holds great promise, as miRNA can now be extracted not only from frozen tumor samples but also from formalin-fixed, paraffin-embedded samples as well as from serum [11]. Expression levels of miR-125b could thus represent a new prognostic marker and candidate predictor of AI response in breast cancer. We aimed to investigate the prognostic value of miR-125b using the miRNA-seq data of The Cancer Genome Atlas (TCGA) breast cancer cohort [79]. However, the insufficient clinical follow-up (low number of relapse events and short median overall follow-up) actually available in this breast cancer cohort does not allow us to make any statements about the prognostic value of miR-125b (data not shown). When a longer median follow-up for the patients in the TCGA cohort who have not yet relapsed is available to the scientific community, it would be of great interest to assess whether high miR-125b expression levels are also found to be associated with shorter RFS in this cohort.

Importantly, reducing miR-125b expression levels in the letrozole-resistant cells was sufficient not only to block the constitutive activation of the AKT/mTOR pathway but also to overcome letrozole resistance, by resensitizing the resistant cells to AI treatment. In the future, therapeutic strategies aimed at blocking expression of miR-125b in endocrine-sensitive primary breast cancer may reinstate a greater response to AI endocrine therapy in a subset of breast cancers and/or may prevent the emergence of miR-125b-overexpressing breast cancer cell subpopulations evolving toward AI-resistant cells.

## Conclusions

Overall, our new findings have important medical implications. (1) The acquisition of specific deregulated miRNAs is a newly discovered alternative mechanism developed by AI-resistant breast cancer cells to achieve constitutive activation of the AKT/mTOR pathway and to develop *de novo* or acquired resistance to AI. (2) MiR-125b, miR-205 and miR-424 represent important players and are similarly deregulated in two independent models of AI resistance. (3) Deregulated expression of these three miRNAs is sufficient to confer AI resistance, activation of the AKT/mTOR pathway and emergence of a subpopulation of cells with stem-like properties. (4) MiR-125b expression levels are informative in breast cancer and may represent a novel biomarker of poor prognosis associated with relapse in ER+ breast cancer patients treated with endocrine therapy, which may ultimately be useful to clinicians in their therapeutic decision-making. (5) Clinical strategies designed to counteract miR-125b-mediated effects could be a valuable future approach to the management of ER+ breast cancers that develop resistance to AI endocrine therapy.

## Additional files

Additional file 1: Table S1. Characteristics of the 65 patients with primary breast cancer from the Centre Léon Bérard (Lyon, France).

Additional file 2: **Supplementary Material and methods.**

Additional file 3: Figure S1. Aromatase inhibitor resistance acquired by the Res-Let is not due to an impaired activity of the aromatase or to an altered ERα expression or functionality.

Additional file 4: Figure S2. RTQ-PCR transfection efficiency validation in Res-Let cells transfected with the inhibitor of miR-125b-5p or the inhibitor of miR-205-5p, the mimic of miR-424-3p, or their respective negative controls.

Additional file 5: Figure S3. Constitutive activation of AKT is sufficient to induce *de novo* resistance to letrozole in MCF-7aro cells.

Additional file 6: Table S2. Statistical comparison of histological grades between tumor samples with low and high miR-125b-5p expression levels.

Additional file 7: Table S3. Statistical comparison of the miR-125b expression levels in the HR+ breast cancer patients treated with endocrine therapy alone or in combination with chemotherapy who had relapsed, or had not, at 7 years.

Additional file 8: Figure S4. miR-125b expression is not deregulated in tamoxifen- or fulvestrant-resistant cells that display no activation of the AKT pathway.

Additional file 9: Figure S5. p53 expression is downregulated, and PI3K p110α is upregulated, when miR-125b is overexpressed in MCF-7aro cells.

## Abbreviations

AD: 4-androstenedione
AI: Aromatase inhibitor
E_2_: 17β-estradiol
ER+: estrogen receptor α-positive
ERα: Estrogen receptor α
FC: Fold change
HER2: Human epidermal growth factor receptor 2
HR+: Hormone receptor–positive (ER+ and/or PR+)
KEGG: Kyoto Encyclopedia of Genes and Genomes
LTED: Long-term estrogen-deprived
MAPK: Mitogen-activated protein kinase
MFE: Mammosphere-forming efficiency
miRNA: microRNA
mTOR: Mammalian target of rapamycin
PI3K: Phosphatidylinositol 3-kinase
PM: Primary mammosphere
PR: Progesterone receptor
RFS: Relapse-free survival
RTQ-PCR: Real-time quantitative PCR
SBR: Scarff-Bloom-Richardson grade
Tam: Tamoxifen
TCGA: The Cancer Genome Atlas
TIC: Tumor-initiating cell
