# Supplementary material for: MicroRNA-125b upregulation confers aromatase inhibitor resistance and is a novel marker of poor prognosis in breast cancer
Source: Breast Cancer Res. 2015 Jan 30;17(1):13. doi: 10.1186/s13058-015-0515-1 (PMC4342894; doi:10.1186/s13058-015-0515-1)
Supplement: Supplementary file 1 — Characteristics of the 65 patients with primary breast cancer from the Centre Léon Bérard (Lyon, France). [file 13058_2015_515_MOESM1_ESM.pdf]

Table S1 Characteristics of the 65 patients with primary breast cancer from the Centre Léon Bérard (Lyon, France).

		Number of patients (n = 65)		
Age (years)				
	≤ 50	29	(44.6%)	44,6
	> 50	36	(55.4%)	55,4
Macroscopic tumor size[a]				
	≤ 30 mm	28	(43.1%)	43,1
	> 30 mm	36	(55.4%)	55,4
Lymph node status				
	≤ 3 involved	40	(61.5%)	61,5
	> 3 involved	25	(38.5%)	38,5
SBR grade[b]				
	1	4	(6.2%)	6,2
	2	18	(27.7%)	27,7
	3	43	(66.2%)	66,2
Estrogen Receptor status[c]				
	Positive	36	(55.4%)	55,4
	Negative	29	(44.6%)	44,6
Progesterone Receptor status[c]				
	Positive	35	(53.8%)	53,8
	Negative	30	(46.2%)	46,2
HER2 status[d]				
	Positive	17	(26.2%)	26,2
	Negative	48	(73.8%)	73,8

[a] Information available for 64 patients.
[b] Scarff-Bloom-Richardson classification.
[c] Measured by immunohistochemistry (IHC).
[d] Measured by IHC. A few samples were validated by Fluorescence *in situ* hybridization (FISH).
