# Supplementary material for: MicroRNA-125b upregulation confers aromatase inhibitor resistance and is a novel marker of poor prognosis in breast cancer
Source: Breast Cancer Res. 2015 Jan 30;17(1):13. doi: 10.1186/s13058-015-0515-1 (PMC4342894; doi:10.1186/s13058-015-0515-1)
Supplement: Supplementary file 2 — Supplementary Material and methods. [file 13058_2015_515_MOESM2_ESM.pdf]

Supplementary Material and Methods

1. Aromatase activity assay

Aromatase activity was determined as previously described [1] using the $^{3}H_2O$ release method. Radioactivity was assessed by liquid scintillation counting and aromatase activity was calculated from dpm as pmol estrogen produced/mg protein/hour.

2. Western-blot

Western-blot experiments were performed as previously described [2]. Antibodies used were: anti-ERα (clone F-10, 1/1000, Santa Cruz Biotechnology, Santa Cruz, CA, USA); anti-phospho-ser167-ERα (clone 16J4, 1/1000), anti-phospho-ser118- ERα (clone D1A3, 1/1000, Cell Signaling, Beverly, MA, USA); anti-α-tubulin (clone DM1A, 1/5000, Sigma), anti-p53 (OP03, 1/1000, Calbiochem, San Diego, CA, USA), anti-PI3K p110α (clone D-4, Santa Cruz Biotechnology).

3. Luciferase assay

Cells were plated and then transfected with an ERE (Estrogen Response Element) firefly luciferase reporter plasmid (ERE-luc) and pTK-RL (Renilla luciferase plasmid). Luciferase activities were assessed as previously described [3].

4. MCF-7aro-myrAkt1 transient transfectants

MCF-7aro cells were transiently transfected using Fugene HD (Roche Diagnostics, Mannheim, Germany) with the myrAkt1 plasmid encoding a constitutively activated form of Akt1 (myrAkt1-transfected MCF-7aro) (1017; Addgene, Cambridge, MA, USA) or the pcDNA6 empty plasmid (pcDNA6-transfected MCF-7aro) (Life technologies) as control.

5. Establishment of tamoxifen- and fulvestrant-resistant cell lines and culture conditions

From the MCF-7aro, two new resistant cell lines denoted Res-Tam and Res-Fulv were established by exposing these MCF-7aro cells during 20 weeks to increasing concentrations of respectively 4-hydroxy-tamoxifen (OH-Tam, 1 and 3 µM) and fulvestrant (0.5 and 1 µM) in Dulbecco’s Modified Eagle Medium without phenol red, supplemented with 3% steroid-depleted, dextran-coated and charcoal-treated fetal calf serum (DCC medium) containing 25 nM 4-androstenedione (AD) (Sigma, St Louis, MO, USA). The OH-Tam, the active metabolite of tamoxifen was from Sigma and the fulvestrant (ICI 182,780) was from Tocris

Cookson (Ellisville, MO). The cells were purged in DCC medium for 4 days prior to each experiment described below. Media and treatment were changed every 2 days.

6. Cytotoxicity assay

MCF-7aro, Res-Tam or Res-Fulv cells were treated for 4 days with 25 nM AD combined with OH-Tam or fulvestrant respectively. Cell viability was assessed as described in the Methods section of the manuscript.

7. Real-time quantitative PCR (RTQ-PCR)

One microgram of total RNA from each sample was reverse-transcribed as described previously [2]. RTQ–PCR measurements were performed using a CFX96 (BioRad, Marne-la-Coquette, France) with the SsoAdvanced Universal SYBR green supermix, according to the manufacturer's recommendations. The forward primer 5'-CCCTTCCCAGAAAACCTACC-3' and the reverse primer 5'-CTCCGTCATGTGCTGTGACT-3' were used to explore the expression of the *TP53* gene. All measurements were normalized to the expression of the *28S* ribosomal gene. Reverse transcription and RTQ-PCR measurements for miR-125b-5p were performed as described in the Methods section of the manuscript.

REFERENCES

1. Cunat S, Rabenoelina F, Daures JP, Katsaros D, Sasano H, Miller WR, Maudelonde T, Pujol P: **Aromatase expression in ovarian epithelial cancers**. *J Steroid Biochem Mol Biol* 2005, **93**(1):15-24.
2. Vendrell JA, Magnino F, Danis E, Duchesne MJ, Pinloche S, Pons M, Birnbaum D, Nguyen C, Theillet C, Cohen PA: **Estrogen regulation in human breast cancer cells of new downstream gene targets involved in estrogen metabolism, cell proliferation and cell transformation**. *J Mol Endocrinol* 2004, **32**(2):397-414.
3. Vendrell JA, Ghayad S, Ben-Larbi S, Dumontet C, Mechti N, Cohen PA: **A20/TNFAIP3, a new estrogen-regulated gene that confers tamoxifen resistance in breast cancer cells**. *Oncogene* 2007, **26**(32):4656-4667.
