# Supplementary material for: MicroRNA-125b upregulation confers aromatase inhibitor resistance and is a novel marker of poor prognosis in breast cancer
Source: Breast Cancer Res. 2015 Jan 30;17(1):13. doi: 10.1186/s13058-015-0515-1 (PMC4342894; doi:10.1186/s13058-015-0515-1)
Supplement: Supplementary file 3 — Aromatase inhibitor resistance acquired by the Res-Let is not due to an impaired activity of the aromatase or to an altered ERα expression or functionality. [file 13058_2015_515_MOESM3_ESM.pdf]

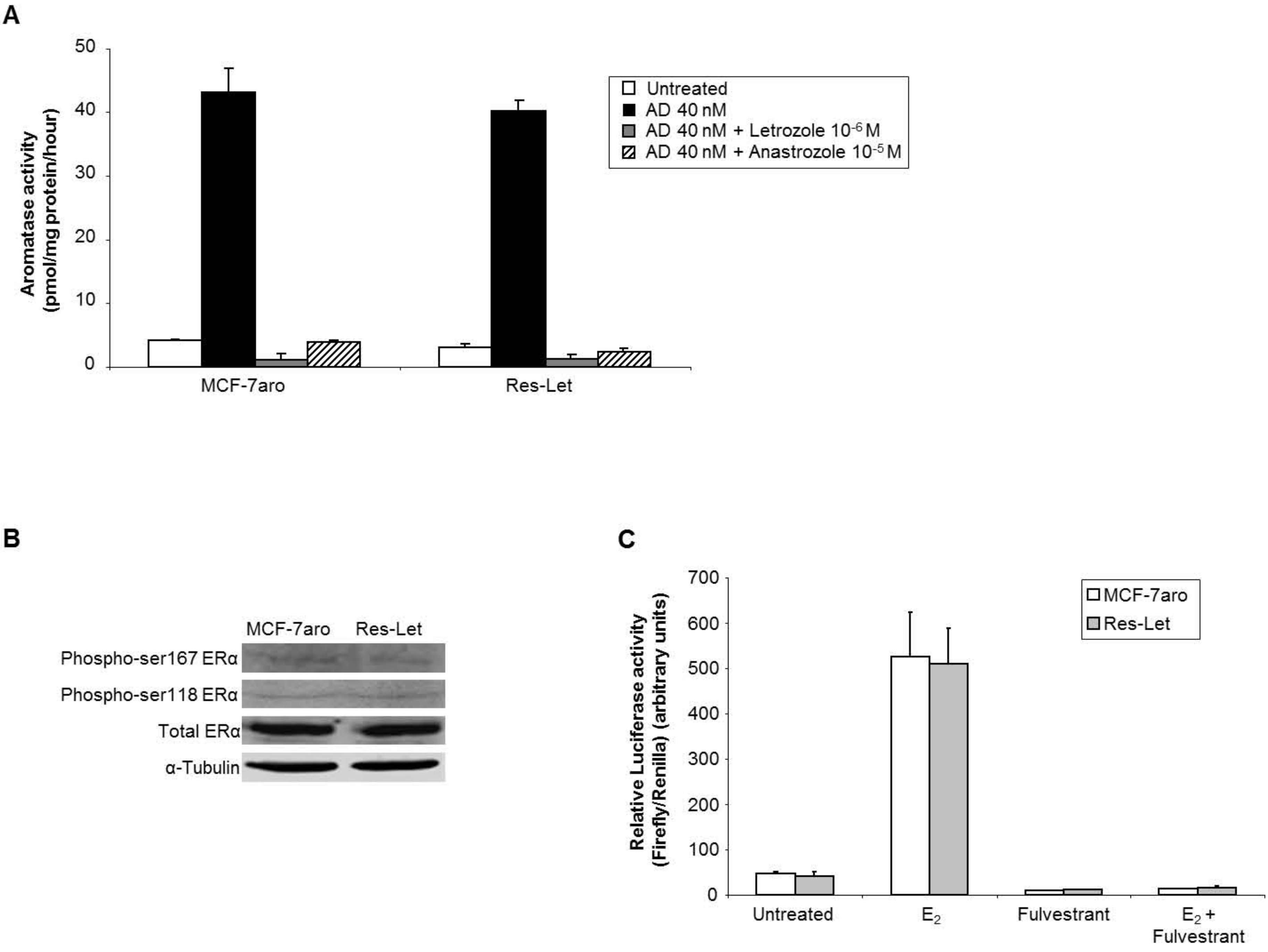

Figure S1 Aromatase inhibitor resistance acquired by the Res-Let is not due to an impaired activity of the aromatase or to an altered ERα expression or functionality. (A) Aromatase activity in MCF-7aro and Res-Let cell lines. AD, 4-androstenedione. **(B)** Western-blot analysis of Phospho-ser167 ERα, phospho-ser118 ERα and total ERα protein expression in MCF-7aro and Res-Let cells. Representative image from three independent cell lysates. **(C)** Relative luciferase activity of MCF-7aro and Res-Let cell lines in response to 17β-estradiol (E_2, 10^{-9} M), fulvestrant (10^{-7} M) or a combination of E_2 + fulvestrant. Histograms represent the ratio of firefly value normalized to renilla value (mean ± SD of three independent experiments).
