# Supplementary material for: MicroRNA-125b upregulation confers aromatase inhibitor resistance and is a novel marker of poor prognosis in breast cancer
Source: Breast Cancer Res. 2015 Jan 30;17(1):13. doi: 10.1186/s13058-015-0515-1 (PMC4342894; doi:10.1186/s13058-015-0515-1)
Supplement: Supplementary file 4 — RTQ-PCR transfection efficiency validation in Res-Let cells transfected with the inhibitor of miR-125b-5p or the inhibitor of miR-205-5p, the mimic of miR-424-3p, or their respective negative controls. [file 13058_2015_515_MOESM4_ESM.pdf]

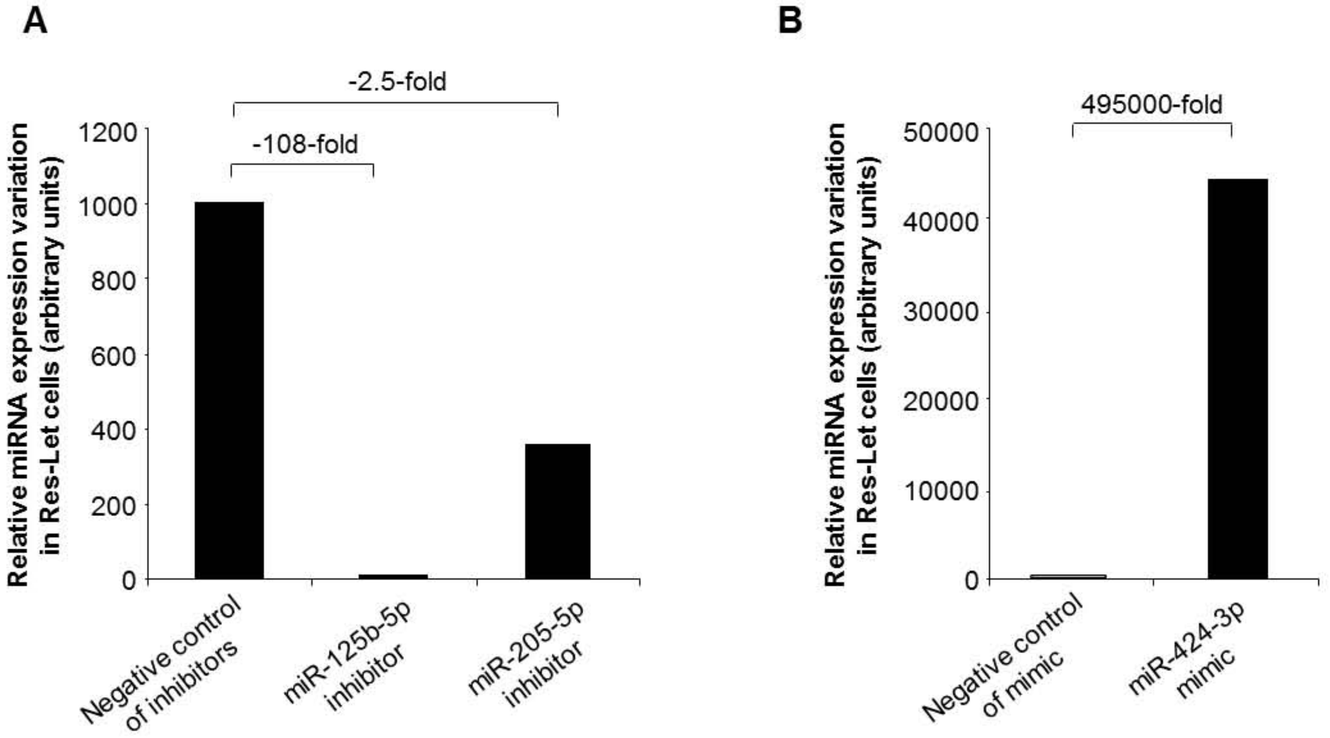

Figure S2 RTQ-PCR transfection efficiency validation in Res-Let cells transfected with the inhibitor of miR-125b-5p or the inhibitor of miR-205-5p, the mimic of miR-424-3p, or their respective negative controls. **(A)** RTQ-PCR transfection efficiency validation in the Res-Let cells transfected with either the negative control of inhibitors, miR-125b-5p inhibitor, miR-205-5p inhibitor or **(B)** with either the negative control of mimic or miR-424-3p mimic (mean ± SD from at least three independent experiments).
