# Supplementary material for: MicroRNA-125b upregulation confers aromatase inhibitor resistance and is a novel marker of poor prognosis in breast cancer
Source: Breast Cancer Res. 2015 Jan 30;17(1):13. doi: 10.1186/s13058-015-0515-1 (PMC4342894; doi:10.1186/s13058-015-0515-1)
Supplement: Supplementary file 5 — Constitutive activation of AKT is sufficient to induce de novo resistance to letrozole in MCF-7aro cells. [file 13058_2015_515_MOESM5_ESM.pdf]

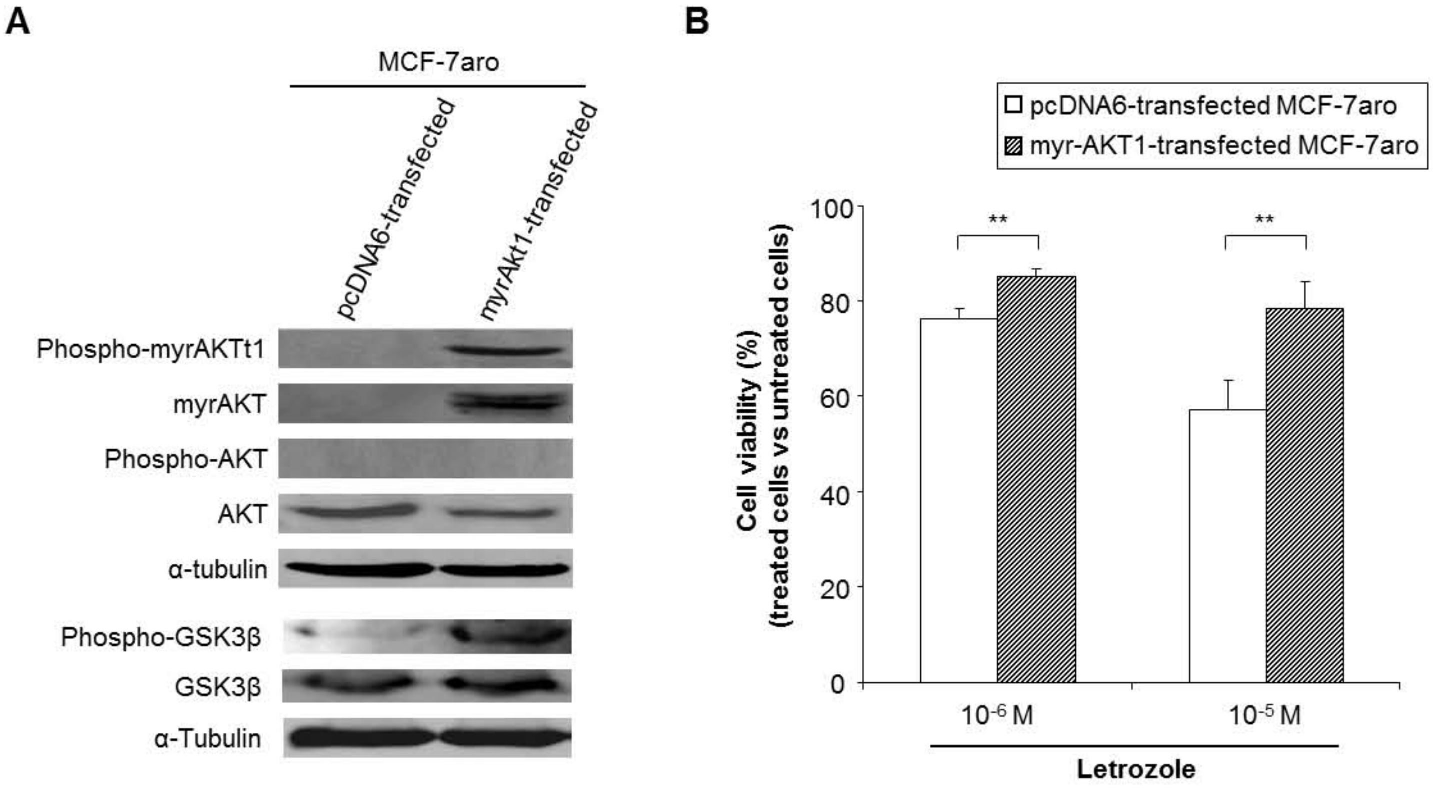

Figure S3 Constitutive activation of AKT is sufficient to induce *de novo* resistance to letrozole in MCF-7aro cells. (A) Western-blot analysis of the activation status of the AKT pathway in MCF-7aro cells transiently transfected by either empty pcDNA6 vector or myrAKT1 vector. **(B)** Cell viability of pcDNA6-transfected MCF-7aro and myrAKT1-transfected MCF-7aro cells in response to letrozole (mean ± SD from four independent experiments). $^{**}P < 0.01$ (Student's *t*-test).
