# Supplementary material for: MicroRNA-125b upregulation confers aromatase inhibitor resistance and is a novel marker of poor prognosis in breast cancer
Source: Breast Cancer Res. 2015 Jan 30;17(1):13. doi: 10.1186/s13058-015-0515-1 (PMC4342894; doi:10.1186/s13058-015-0515-1)
Supplement: Supplementary file 6 — Statistical comparison of histological grades between tumor samples with low and high miR-125b-5p expression levels. [file 13058_2015_515_MOESM6_ESM.pdf]

Table S2 Statistical comparison of the different histological grades between tumor samples with low and high miR-125b-5p expression levels

		Number of samples (%)		
		**Low miR-125b-5p expression levels**	**High miR-125b-5p expression levels**	***P*[a]**
Histological grade				
	SBR1	1 (11%)	3 (23%)	0.62
	SBR2	8 (89%)	10 (77%)	
Histological grade				
	SBR1	1 (4%)	3 (14%)	0.33
	SBR3	24 (96%)	19 (86%)	
Histological grade				
	SBR2	8 (25%)	10 (34%)	0.57
	SBR3	24 (75%)	19 (66%)	
Histological grade				
	SBR1+2	9 (27%)	13 (40%)	0.30
	SBR3	24 (73%)	19 (60%)	

[a]*P* (Fisher exact test) was considered significant when $P < 0.05$.
