# Supplementary material for: MicroRNA-125b upregulation confers aromatase inhibitor resistance and is a novel marker of poor prognosis in breast cancer
Source: Breast Cancer Res. 2015 Jan 30;17(1):13. doi: 10.1186/s13058-015-0515-1 (PMC4342894; doi:10.1186/s13058-015-0515-1)
Supplement: Supplementary file 7 — Statistical comparison of the miR-125b expression levels in the HR+ breast cancer patients treated with endocrine therapy alone or in combination with chemotherapy who had relapsed, or had not, at 7 years. [file 13058_2015_515_MOESM7_ESM.pdf]

Table S3 Statistical comparison of the miR-125b expression levels in the HR+ breast cancer patients treated with endocrine therapy alone or combined to chemotherapy who relapsed or not at 7 years (*n* = 11).

	**Number of patients (%)**		
	**Patients who did not relapse (n = 7)**	**Patients who relapsed (n = 4)**	***P*[a]**
miR-125b-5p expression levels			
Low[b]	7 (100%)	1 (25%)	0.02
High	0 (0%)	3 (75%)	

[a]*P* (Fisher exact test) was considered significant when $P < 0.05$.
[b] the median value was used to dichotomise the cohort into two groups with either low or high miR-125b-5p expression levels.
