# Supplementary material for: MicroRNA-125b upregulation confers aromatase inhibitor resistance and is a novel marker of poor prognosis in breast cancer
Source: Breast Cancer Res. 2015 Jan 30;17(1):13. doi: 10.1186/s13058-015-0515-1 (PMC4342894; doi:10.1186/s13058-015-0515-1)
Supplement: Supplementary file 8 — miR-125b expression is not deregulated in tamoxifen- or fulvestrant-resistant cells that display no activation of the AKT pathway. [file 13058_2015_515_MOESM8_ESM.pdf]

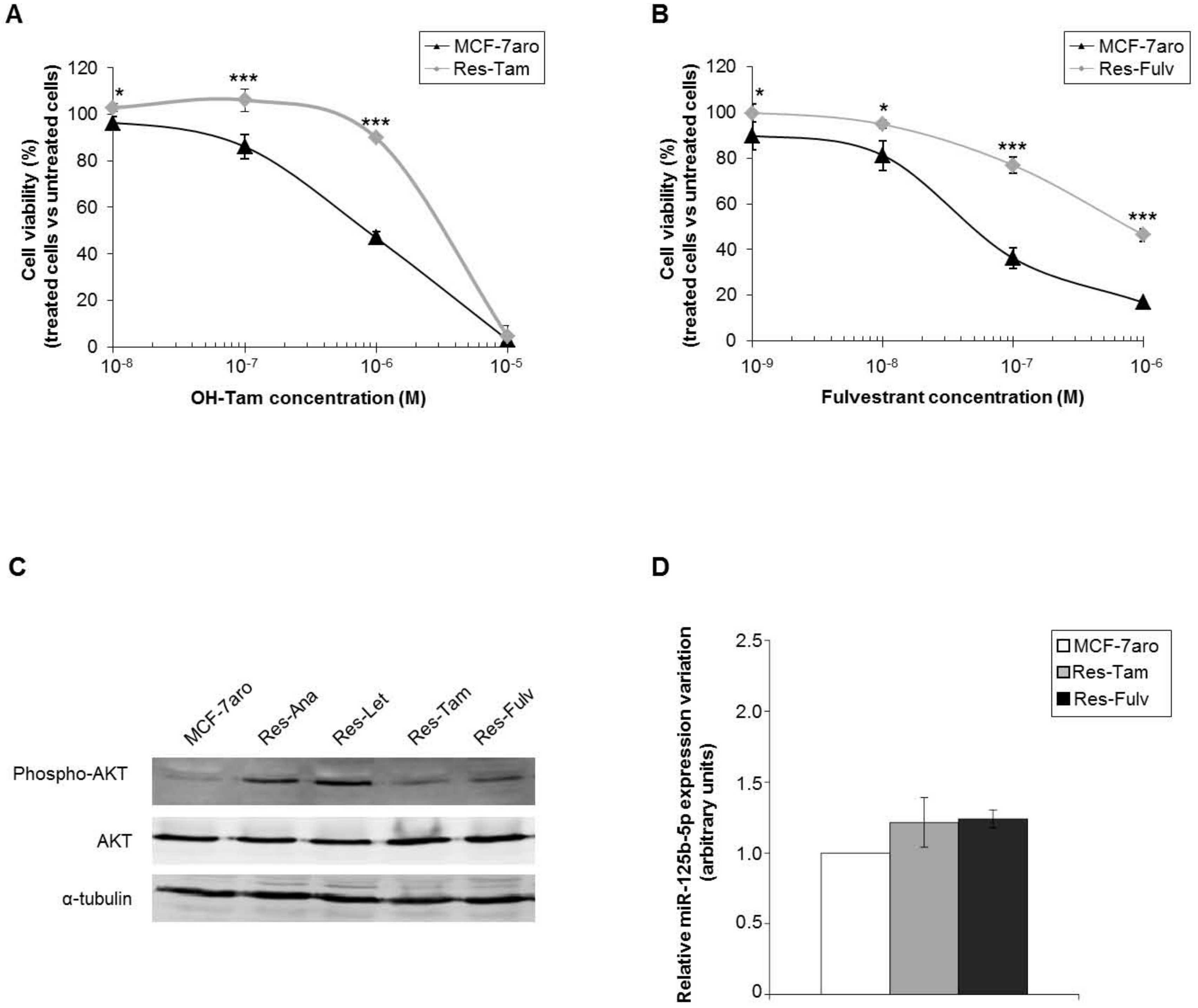

Figure S4 miR-125b expression is not deregulated in tamoxifen or fulvestrant resistant cells that display no activation of the AKT pathway. Pharmacological response of MCF-7aro and Res-Tam cells to OH-Tam **(A)** and of MCF-7aro and Res-Fulv cells to fulvestrant **(B)** as assessed by cytotoxicity assay (mean ± SD from three independent experiments). *$P < 0.05$, and ***$P < 0.001$ versus the corresponding MCF-7aro (Student's *t*-test). **(C)** Western-blot analysis of the AKT status in MCF-7aro, Res-Ana, Res-Let, Res-Tam and Res-Fulv cells. Representative image from three independent experiments and cell lysates. **(D)** RTQ-PCR analysis of miR-125b-5p expression levels in Res-Tam and Res-Fulv cells versus MCF-7aro cells (mean ± SD from at least three independent experiments).
