# Supplementary material for: MicroRNA-125b upregulation confers aromatase inhibitor resistance and is a novel marker of poor prognosis in breast cancer
Source: Breast Cancer Res. 2015 Jan 30;17(1):13. doi: 10.1186/s13058-015-0515-1 (PMC4342894; doi:10.1186/s13058-015-0515-1)
Supplement: Supplementary file 9 — p53 expression is downregulated, and PI3K p110α is upregulated, when miR-125b is overexpressed in MCF-7aro cells. [file 13058_2015_515_MOESM9_ESM.pdf]

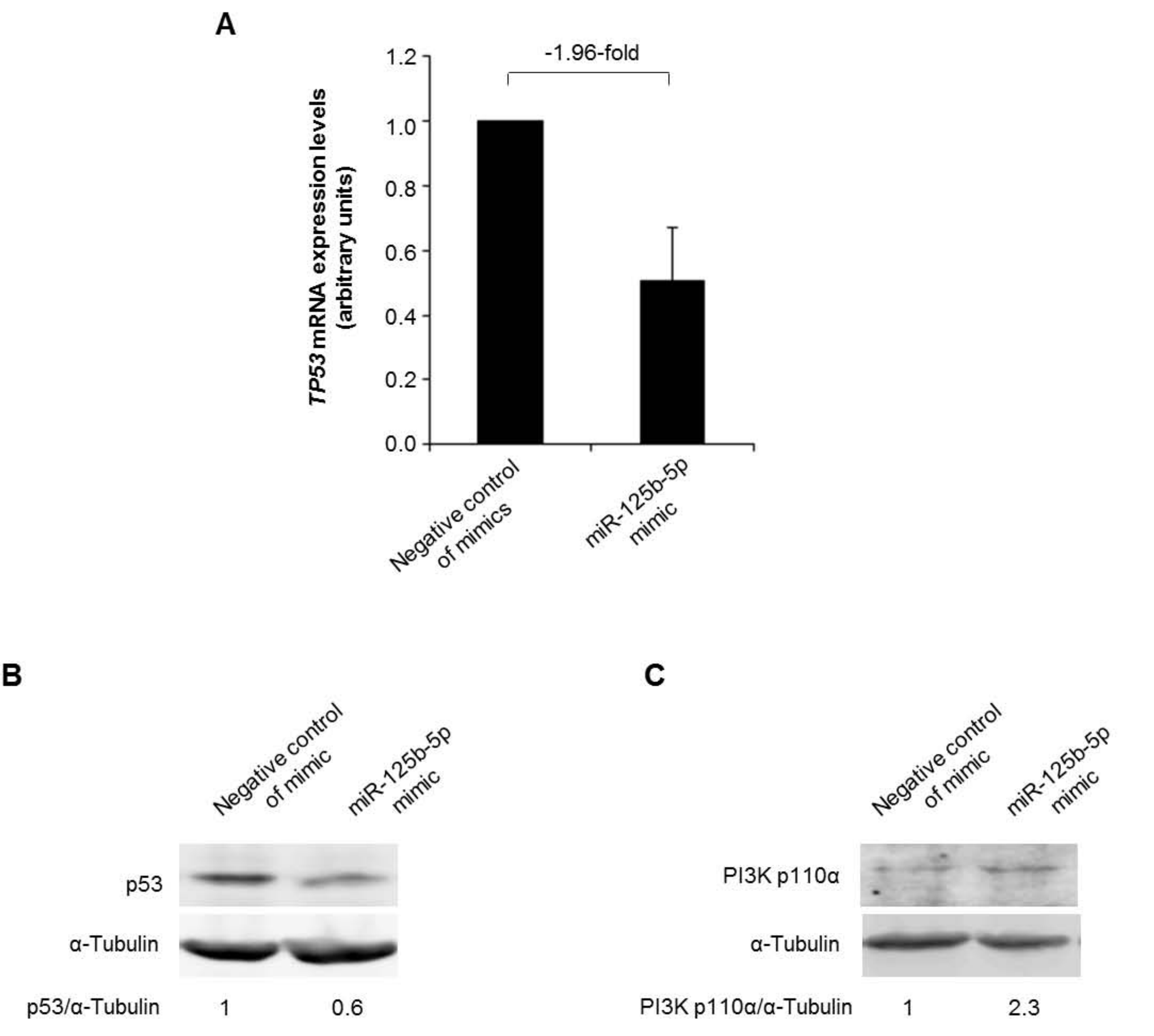

Figure S5 p53 expression is down-regulated and PI3K p110α is up-regulated when miR-125b is over-expressed in MCF-7aro cells. (A) RTQ-PCR validation of *TP53* deregulated expression levels in MCF-7aro cells transfected with the mimic of miR-125b-5p or the negative control (mean ± SD from two independent experiments). **(B)** Western-blot analysis of p53 and **(C)** of PI3K p110α in MCF-7aro cells transfected with the mimic of miR-125b-5p or the negative control. Representative images from two independent experiments and cell lysates. The relative expression levels of the corresponding ratio p53 / α-Tubulin or PI3K p110α / α-Tubulin were quantified and shown at the bottom of the panels.
